# Controllable site-selective C–C bond cleavage for the divergent synthesis of imidazo[1,5-*a*]pyridine derivatives

**DOI:** 10.1039/d5ra07797d

**Published:** 2025-12-10

**Authors:** Qiang Wang, Xinyu Song, Shujun Qin, Wenya Dong, Pingjing Xue, Jiayi Wu, Haibo Wang

**Affiliations:** a College of Biomedical and Health Sciences, Anhui Science and Technology University Fengyang 233100 PR China wangqiang@ahstu.edu.cn

## Abstract

Ethyl 3-oxo-3-phenylpropanoate reacts with phenyl(pyridin-2-yl)methanamine to afford a selective product *via* the site-selective C–C bond cleavage of ethyl 3-oxo-3-phenylpropanoate by adjusting reaction parameters, and a series of imidazo[1,5-*a*]pyridines can be effectively prepared using this method. This study enables controllable site-selectivity in both C–C bond cleavage and C–C bond formation, which may offer new insights for chemists in the C–C bond cleavage research field.

## Abstract 2

Ethyl 3-oxo-3-phenylpropanoate reacts with phenyl(pyridin-2-yl)methanamine to afford a selective product *via* the site-selective C–C bond cleavage of ethyl 3-oxo-3-phenylpropanoate by adjusting reaction parameters.

## Graphical abstract

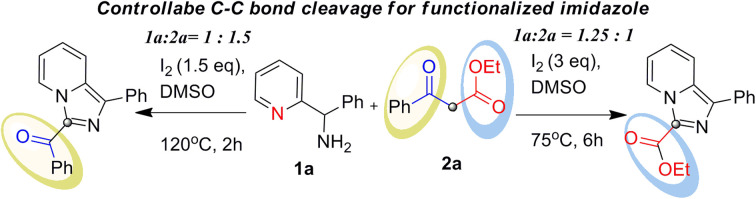

## Introduction

Organic reactions mediated by C–C bond cleavage have attracted the attention of many researchers in recent years, but compared with conventional C–C bond formation reactions, this area is still in the early stages and remains to be explored.^1–4^ It is evident from the literature that there are three challenges in organic reactions mediated by C–C bond cleavage.^1,3,5–13^ Firstly, reactions mediated by C–C bond cleavage are not as extensive as C–C bond formation reactions, and there is no mature theory to guide chemists to apply such reactions. The second problem is the selectivity of C–C bond cleavage, that is, the selectivity between the retained carbon group and the leaving carbon group is difficult to achieve when the C–C bond breaks. To be specific, if the electronic and spatial properties of the bonding carbon group and the leaving carbon group are significantly different, the selectivity is relatively easy to achieve, otherwise, there are greater difficulties. The third problem is the controllability of C–C bond cleavage for directional synthesis. Theoretically, it is possible to realize the switch between the bonding group and the leaving group by adjusting the experimental conditions, while related study is rarely reported.

**Scheme 1:**
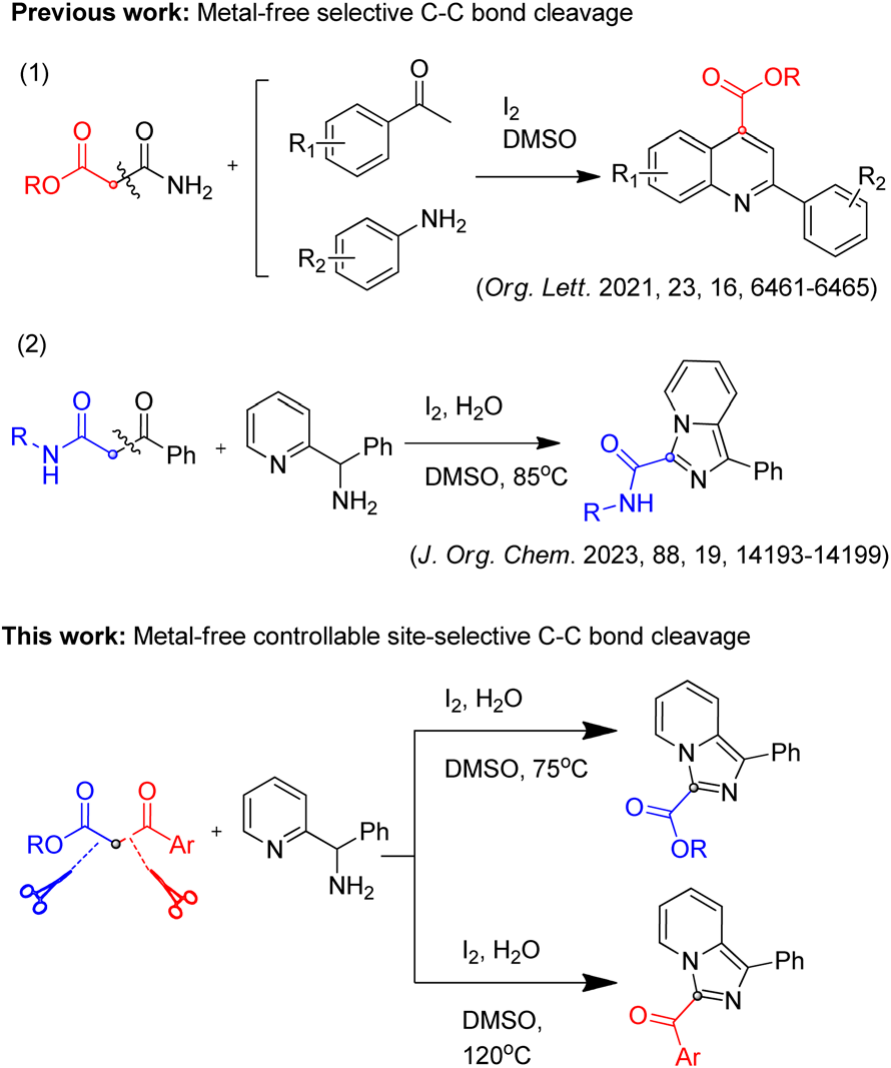
Selective C–C bond cleavage and controllable site-selective C–C bond cleavage mediated reactions.

1,3-Dicarbonyl compounds are important synthons widely used in various synthetic reactions, and their selective C–C bond cleavage reactions have been also reported in several papers.^8,14–18^ These reactions mainly involve transition metal catalyzed C–C bond cleavage mediated bond formation. Besides, only two studies of metal-free selective C–C bond cleavage mediated for heterocycles synthesis have been reported^17,19^ (Scheme 1), which makes it possible to synthesize carbonyl-derived heterocycles by using 1,3-dicarbonyl compounds as a carbonyl source. Compared with single selectivity of C–C cleavage reactions of 1,3-dicarbonyl compounds above, controllable C–C cleavage reactions at two sites for two different carbonyl derived products have not been reported to the best of our knowledge. 1,3-dicarbonyl compounds are good model substrates, due to the methylene group being connected to two carbon groups, which have similar electronic and spatial properties on both sides, whether the cleavage site could be controlled is worth trying.

Based on the reported work and our previous studies,^19^ we conducted deep exploration and realized controllable two-site C–C cleavage mediated reaction of 1,3-dicarbonyl compounds by adjusting the reaction conditions, and successfully synthesized 3-ester imidazo[1,5-*a*]pyridines and 3-acyl imidazo[1,5-*a*]pyridines, respectively (Scheme 1). Such compounds have a wide range of biological activities in the field of medicinal chemistry,^20–22^ therefore, this method is very useful for medicinal chemists in related areas.

## Results and discussion

As shown in Table 1, phenyl(pyridin-2-yl)methanamine (1a) and ethyl 3-oxo-3-phenylpropanoate (2a) and were used as model substrates to explore the controllability of C–C bond cleavage. The results showed that substrate ratio, the amount of I_2_, temperature and reaction time all affected the reaction results by selecting C–C cleavage sites. With 1a and 2a in equimolar amounts (0.2 mmol : 0.2 mmol), 3 equivalents of I_2_, and the reaction conducted in DMSO at different temperatures, it was found that lower temperature favored the C–C bond cleavage at the benzoyl group side and provided 3-ester imidazo[1,5-*a*] pyridine (3a′) as single product (entries 1 and 2), with the temperature increased, the C–C bond cleavage site gradually transferred to the ester side and provided 3-benzoyl imidazo[1,5-*a*] pyridine (3a) and 3-ester imidazo[1,5-*a*] pyridine (3a′) (entries 1, 2 *vs.* entries 3–5), and the reaction time was greatly saved to 3 hours (entry 5) and 2 hours (entries 6 and 8–13), also the yield of 3a′ was increased with temperature increasing (entry 5 *vs.* entries 6 and 8–13). When the temperature was 120 °C, 3-benzoyl imidazo[1,5-*a*] pyridine (3a) presented as major product (56%) and primary selectivity on another side could be observed (entry 6), these results indicated that temperature was a key factor. With the phenomenon observed, further investigation for controllable C–C bond cleavage at ester side was performed. Firstly, adjusting substrate ratio to 0.25 : 0.2, and keep the other factors unchanged, satisfactory yield (76%) of 3a′ could be obtained at 75 °C as single product (entry 7). Keeping this substrate ratio and gradually adjusting the amount of I_2_ provided elevated selectivity for the other product (3a) with modest yields at 120 °C (entries 8–9), while reducing I_2_ to 0.2 mmol the yield was affected greatly (entry 10). Subsequently, adjusting the substrate ratio to 0.2 : 0.25 and 0.2 : 0.3, satisfactory selectivity results were obtained (entries 11–13), 3a (75%) and 3a′ (7%) were obtained in entry 12, more than 10 : 1 selectivity was obtained, and the reaction was not optimized further.

**Table 1:** Effects of reaction parameters^a^

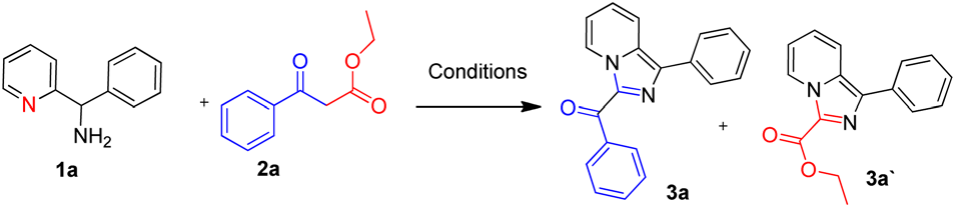						
Entry	1a : 2a (mmol)	I_2_ (mmol)	*T* (°C)	Reaction time (h)	Yield	
					3a	3a′
1	0.2 : 0.2	0.6	70	6	n.d	51%
2	0.2 : 0.2	0.6	75	6	n.d	56%
3	0.2 : 0.2	0.6	80	6	29%	41%
4	0.2 : 0.2	0.6	100	6	43%	32%
5	0.2 : 0.2	0.6	110	3	50%	23%
6	0.2 : 0.2	0.6	120	2	56%	18%
7	0.25 : 0.2	0.6	75	6	n.d	76%
8	0.25 : 0.2	0.4	120	2	57%	13%
9	0.25 : 0.2	0.3	120	2	64%	10%
10	0.25 : 0.2	0.2	120	2	43%	7%
11	0.2 : 0.25	0.3	120	2	69%	10%
12	0.2 : 0.3	0.3	120	2	75%	7%
13	0.2 : 0.4	0.3	120	2	61%	6%

a Reaction conditions: specified amount of phenyl(pyridin-2-yl)methanamine (1a), ethyl 3-oxo-3-phenylpropanoate (2a), I_2_ and H_2_O (0.6 mmol) were stirred with specified temperature and reaction time in DMSO (2 mL). Isolated yields were shown. n.d. = not detected.

With the optimized conditions in hand, a series of 1,3-dicarbonyl compounds were employed to investigate the applicability of this controllable C–C bond cleavage mediated reaction. As shown in Table 2, derivatives of ethyl 3-oxo-3-phenylpropanoate were firstly examined. Electron-donating group methyl at *ortho*-position, *meta*-position and *para*-position all provided the target product 3a′ with 73–76% yields under conditions of 75 °C, and exhibited excellent selectivity without detection of 3-acyl imidazo[1,5-*a*] pyridine. Unsurprisingly, at 120 °C, these three substrates still maintained high selectivity, exhibiting similar selectivity profiles and yields to those of 2a and gave products 3b, 3c and 3d (entries 1–3). Similarly, electron-withdrawing group bromide at *ortho*-position, *meta*-position and *para*-position all provided the target product 3a′ as well with 72–76% yields under conditions of 75 °C, and provided 3e, 3f and 3g (76–79%) under the conditions of 120 °C (entries 4–6). It was found that electron-withdrawing group and electron-donating group both provided similar selectivity profile and yields at 75 °C and 120 °C, indicating electronic effects did not significantly affect the reaction results. Other common substituents were also examined under the optimized conditions of 75 °C and 120 °C respectively, such as halogen group, fluorine, chloride, and iodine substituents all reacted smoothly and provided similar selectivity profile under the optimized condition (entries 7–9). Electron-donating group, methoxy group and other electron-withdrawing group –CF_3_, –NO_2_ and –CN all provided similar results (entries 10–13). Besides, multi-substituted ethyl 3-oxo-3-phenylpropanoate derivatives were also investigated and the results were similar as that of 2a (entries 15–17). Moreover, heteroaromatic ring derived 1,3-dicarbonyl compounds, methyl ester and *n*-butyl ester 1,3-dicarbonyl compounds were still applicable under the optimized condition and exhibited similar selectivity profile (entries 18–20). Results above demonstrated that this controllable C–C bond cleavage reaction was applicable for various analogues of ethyl 3-oxo-3-phenylpropanoate. In addition to the reaction results listed in the Table 2, we also examined 1a analogues 1-(pyridin-2-yl)ethanamine and pyridin-2-ylmethanamine reacting with ethyl 3-oxo-3-phenylpropanoate, but the target products were not detected under 75 °C, and the reaction system were rather chaotic at 120 °C.These results indicated that the benzene ring of phenyl(pyridin-2-yl)methanamine was essential to the reaction.

**Table 2:** Investigation on various 1,3-dicarbonyl compounds for controllable C–C cleavage reactions

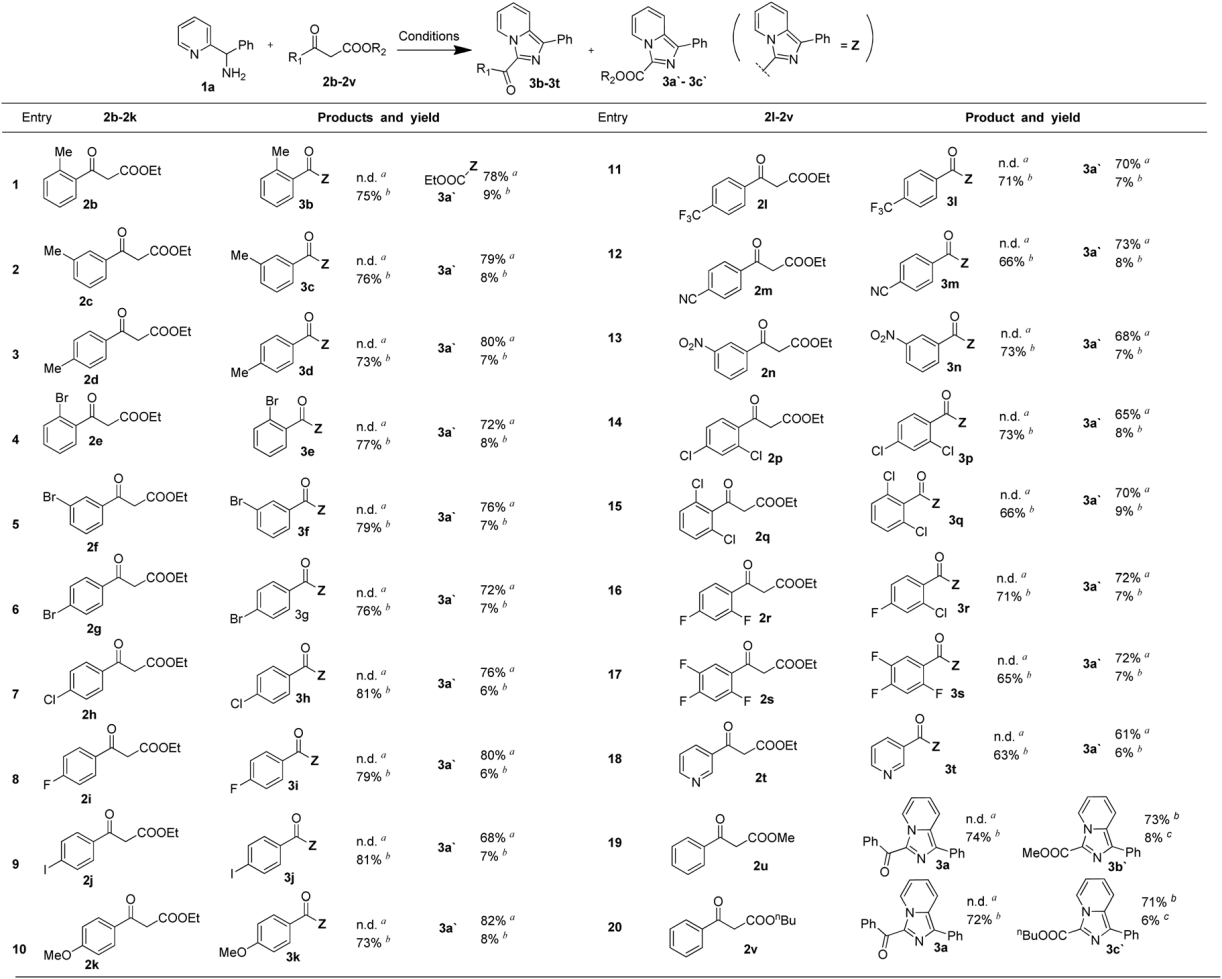

a Conditions: reactions were carried out using 1,3-dicarbonyl compounds (0.2 mmol), phenyl(pyridin-2-yl)methanamine (0.25 mmol), I_2_ (0.6 mmol) and H_2_O (0.6 mmol) in 2 mL of DMSO and stirred at 75 °C for 6 h.

b Reactions were carried out using 1,3-dicarbonyl compounds (0.3 mmol), phenyl(pyridin-2-yl)methanamine (0.2 mmol), I_2_ (0.3 mmol) and H_2_O (0.6 mmol) in 2 mL of DMSO and stirred at 120 °C for 2 h.

**Scheme 2:**
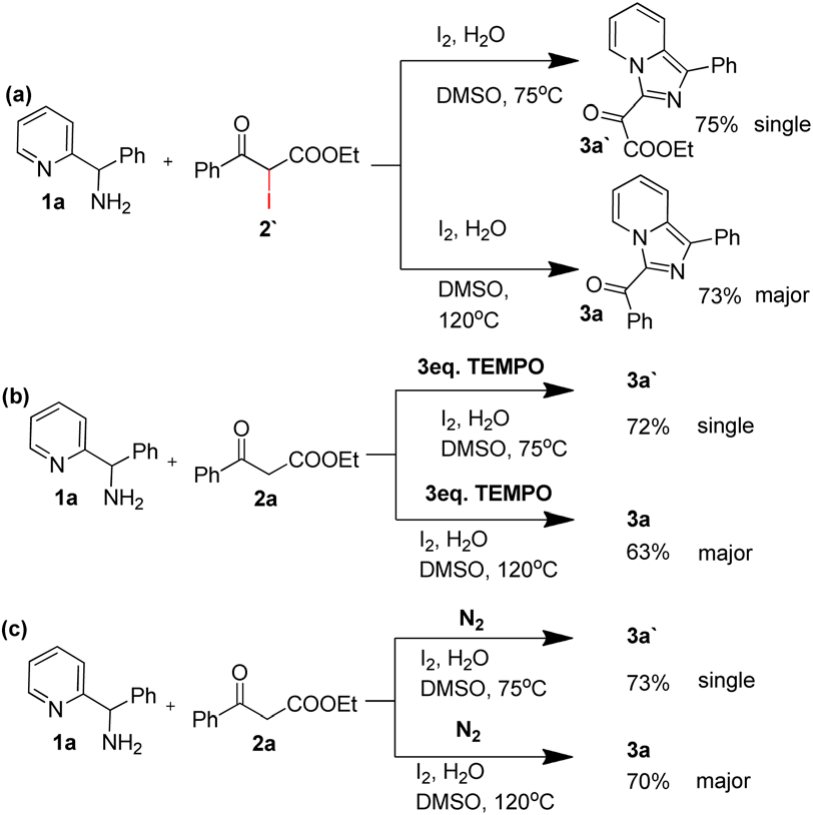
Control experiments.

To obtain more information about the reaction mechanism, a set of control experiments were performed. As shown in Scheme 2, ethyl 2-iodo-3-oxo-3-phenylpropanoate reacted with phenyl(pyridin-2-yl)methanamine under two standard conditions could provide target product 3a with 77% yield and 3a′ with 73% yield respectively (Scheme 2a). Besides, 2,2,6,6-tetramethylpiperidoxyl (TEMPO) had little effect on the yield under the standard conditions, indicating this reaction did not experience free radical process (Scheme 2b). Moreover, oxygen free experiments demonstrated that oxygen was unnecessary in this transformation (Scheme 2c).

**Scheme 3:**
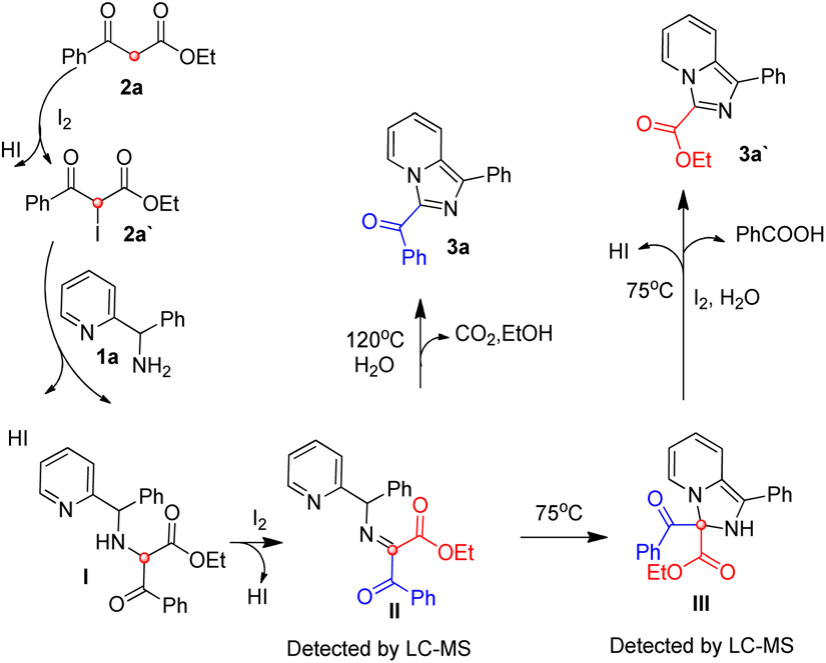
Possible mechanism.

Based on our previous reports^17,19^ and the experimental results, a possible mechanism was depicted in Scheme 3. 2a is iodized on the methylene to form 2a′, 2a′ reacts with 1a*via* nucleophilic reaction to form intermediate I, which is oxidized by iodine to give intermediate II. At 75 °C, intermediate II could experience an intramolecular nucleophilic attack to give intermediate III, intermediate III experience a iodine and water mediated elimination and dehydrogenation to provide 3a′ and benzoic acid. At 120 °C, Intermediate II mainly experience an intramolecular nucleophilic substitution and provide 3a, carbon dioxide and ethanol directly, and a small part of intermediate II unavoidably experience the reaction process of 75 °C to produce minor product 3a′.

**Scheme 4:**
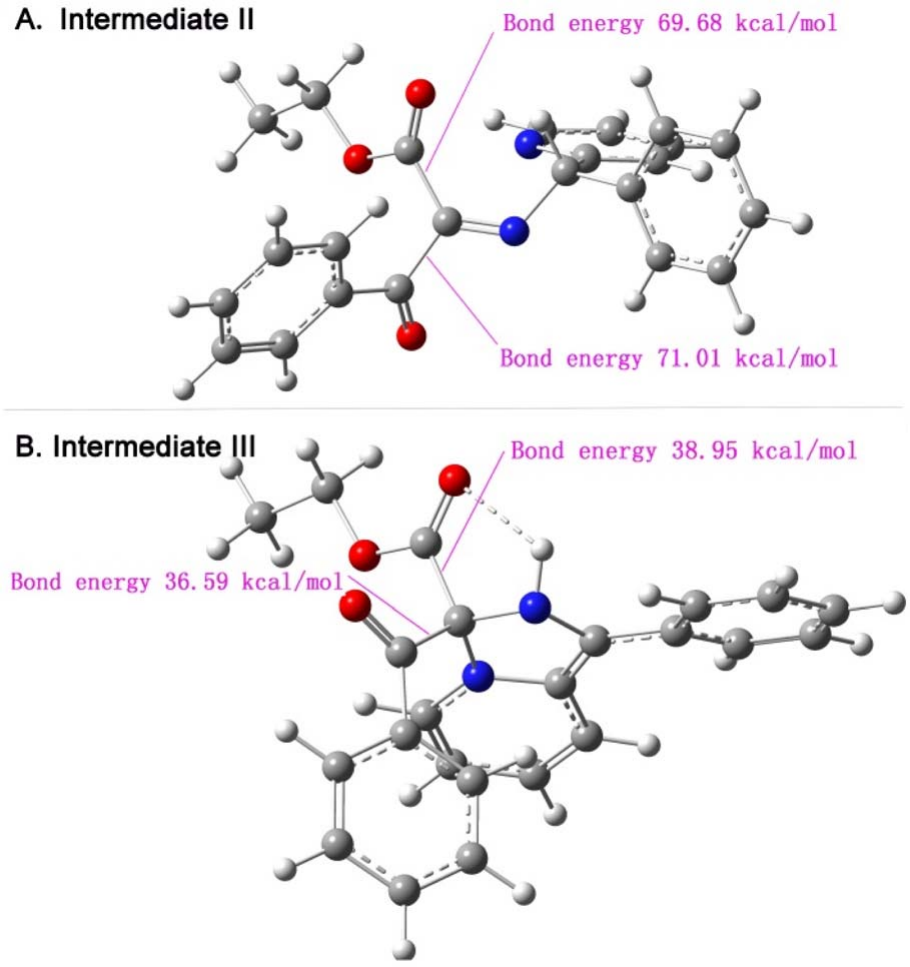
Gaussian calculation of bond energy of intermediate II and III.

The proposed mechanism was also supported in the further Gaussian calculation modeling, Comparison of the C-ester bond energy and C-benzoyl bond energy in intermediates II and III provides theoretical support for the proposed mechanism. As shown in scheme 4A, the C-ester bond energy (69.68 kcal mol^−1^) is less than C-benzoyl bond energy (71.01 kcal mol^−1^), II tends to preferentially remove the ester group under the intramolecular nucleophilic attack at 120 °C. When the reaction temperature is 75 °C, II transformed to III, and the C-ester bond energy and C-benzoyl bond energy in III both decreased significantly (Scheme 4B). Besides, intramolecular hydrogen bond formation made C-ester bond energy (38.95 kcal mol^−1^) higher than C-benzoyl bond energy (36.59 kcal mol^−1^). These results provided a relatively rational explanation of experimental phenomenon.

## Conclusions

In conclusion, an iodine-mediated controllable site-selective C–C bond cleavage of ethyl 3-oxo-3-phenylpropanoate has been achieved for the preparation of 3-carbonyl-substituted imidazo[1,5-*a*]pyridines. This study provides an alternative method for the efficient synthesis of 3-carbonyl-substituted imidazo[1,5-*a*]pyridines with satisfactory yields. More importantly, this study has for the first time achieved controllable site-selective C–C bond cleavage of 1,3-dicarbonyl compounds by simply adjusting reaction conditions, and a relatively reasonable reaction mechanism supported by both experimental and theoretical calculation results has been proposed.

## Experimental section

### Reagents and instruments

Unless otherwise noted, all reactions were performed under an air atmosphere. Reagents were purchased from the Tansoole reagent platform and used directly. ^1^H NMR and ^13^C NMR spectra were measured at room temperature. High-resolution mass spectra (HRMS) were obtained from Waters UPLC G2-XS QTOF. Flash column chromatography was performed on 200–300 mesh of silica gel.

### General procedure for the controllable site-selective C–C bond cleavage mediated reactions

#### Method A (for products 3a′, 3b′ and 3c′)

To a solution of 1,3-dicarbonyl compound (0.2 mmol, 1 equiv.), pyridine ethylamine (0.25 mmol, 1.25 equiv.) in 2 mL of DMSO in a tube was added I_2_ (0.6 mmol, 3 equiv.) and H_2_O (0.6 mmol), and the tube was heated at 75 °C and for 6 hours with oil bath. The system was cooled to room temperature, water (10 mL) was added and the aqueous solution was extracted with ethyl acetate (3 × 30 mL), the combined organic phase was washed with saturated brine and dried over anhydrous Na_2_SO_4_. The organic phase was concentrated under reduced pressure, residue was chromatographed through silica gel eluting with ethyl acetate/petroleum ether to give the product.

#### Method B (for products 3a–3t)

To a solution of 1,3-dicarbonyl compound (0.3 mmol, 1.5 equiv.), pyridine ethylamine (0.2 mmol, 1 equiv.) in 2 mL of DMSO in a tube was added I_2_ (0.3 mmol, 1.5 equiv.) and H_2_O (0.6 mmol), and the tube was heated at 120 °C and for 2 hours with oil bath. The system was cooled to room temperature, water (10 mL) was added and the aqueous solution was extracted with ethyl acetate (3 × 30 mL), the combined organic phase was washed with saturated brine and dried over anhydrous Na_2_SO_4_. The organic phase was concentrated under reduced pressure, residue was chromatographed through silica gel eluting with ethyl acetate/petroleum ether to give the product.

### Gaussian calculation of bond energy of intermediates II and III

The B3LYP^23^ density functional method with the D3(BJ)^24^ dispersion correction was employed in this work to carry out all the computations. The def2 TZVP basis^25^ set was used for the atoms in geometry optimizations using the PCM model^26^ with DMSO as the solvent. Vibrational frequency analyses at the same level of theory were performed to characterize stationary points as local minima without any imaginary frequencies. All DFT theoretical calculations have been carried out using the Gaussian program package.

### NMR spectra data for products

#### Phenyl(1-phenylimidazo[1,5-*a*]pyridin-3-yl)methanone (3a)^27^

According to method B and purified by column chromatography with ethyl acetate/petroleum ether (v/v = 1 : 4) to obtain yellow solid ( 44.7 mg, 75%). ^1^H NMR (600 MHz, CDCl_3_) *δ* 9.93 (d, *J* = 7.1 Hz), 8.60–8.49 (m), 8.08 (d, *J* = 9.0 Hz), 8.00–7.94 (m), 7.63–7.57 (m), 7.56–7.48 (m), 7.43–7.35 (m), 7.28 (ddd, *J* = 8.9, 6.6, 0.9 Hz), 7.11–7.04 (m). ^13^C (151 MHz, CDCl_3_) *δ* 182.2, 138.2, 134.7, 134.0, 133.9, 132.1, 131.2, 130.9, 128.8, 128.0, 127.6, 127.3, 125.1, 118.4, 116.4.

#### (1-Phenylimidazo[1,5-*a*]pyridin-3-yl)(*o*-tolyl)methanone (3b)^27^

According to method B and purified by column chromatography with ethyl acetate/petroleum ether (v/v = 1 : 4) to obtain yellow solid (46.8 mg, 75%). ^1^H NMR (600 MHz, CDCl_3_) *δ* 9.92 (d, *J* = 7.2 Hz), 8.47 (d, *J* = 8.2 Hz), 8.06 (d, *J* = 9.0 Hz), 7.98–7.95 (m), 7.51 (t, *J* = 7.7 Hz), 7.41–7.33 (m), 7.27–7.25 (m), 7.07–7.03 (m), 2.46 (s). ^13^C NMR (151 MHz, CDCl_3_) *δ* 182.0, 153.4, 142.8, 135.5, 134.4, 134.1, 134.0, 131.0, 128.8, 128.7, 127.5, 127.3, 124.9, 118.3, 116.2.

#### (1-Phenylimidazo[1,5-*a*]pyridin-3-yl)(*m*-tolyl)methanone (3c)^27^

According to method B and purified by column chromatography with ethyl acetate/petroleum ether (v/v = 1 : 4) to obtain yellow solid (47.4 mg, 76%). ^1^H NMR (600 MHz, CDCl_3_) *δ* 9.92 (d, *J* = 7.1 Hz), 8.37 (d, *J* = 7.4 Hz), 8.26 (s), 8.07 (d, *J* = 9.0 Hz), 7.99–7.93 (m), 7.51 (t, *J* = 7.7 Hz), 7.45–7.36 (m), 7.28 (ddd, *J* = 8.9, 6.6, 0.6 Hz), 7.10–7.03 (m), 2.48 (s). ^13^C NMR (151 MHz, CDCl_3_) *δ* 182.5, 138.1, 137.6, 134.5, 134.0, 134.0, 132.9, 131.2, 128.8, 128.4, 127.8, 127.5, 127.3, 125.0, 118.4, 116.4, 21.5.

#### Ethyl 1-phenylimidazo[1,5-*a*]pyridine-3-carboxylate(3a′)^19^

According to method A and purified by column chromatography with ethyl acetate/petroleum ether (v/v = 1 : 4) to obtain pale oil (yields shown in Table 1 entries 1–18 for each reaction). ^1^H NMR (400 MHz, CDCl_3_) *δ* 9.41 (d, *J* = 7.2 Hz, 1H), 8.00–7.86 (m, 3H), 7.47 (t, *J* = 7.6 Hz, 2H), 7.35 (t, *J* = 7.4 Hz, 1H), 7.16–7.07 (m, 1H), 6.95 (dd, *J* = 10.0, 3.7 Hz, 1H), 4.55 (q, *J* = 7.1 Hz, 2H), 1.49 (t, *J* = 7.1 Hz, 3H). ^13^C NMR (101 MHz, CDCl_3_) *δ* 159.8, 134.0, 133.7, 130.7, 128.69, 127.5, 127.4, 126.6, 125.9, 123.1, 118.5, 115.5, 61.3, 14.5.

#### Methyl 1-phenylimidazo[1,5-*a*]pyridine-3-carboxylate(3b′)^19^

According to method A and purified by column chromatography with ethyl acetate/petroleum ether (v/v = 1 : 4) to obtain yellow solid (36.8 mg, 73%). ^1^H NMR (400 MHz, CDCl_3_) *δ* 9.40 (d, *J* = 7.2 Hz, 1H), 7.98 (d, *J* = 9.1 Hz, 1H), 7.90 (d, *J* = 7.6 Hz, 2H), 7.48 (t, *J* = 7.6 Hz, 2H), 7.36 (t, *J* = 7.4 Hz, 1H), 7.14 (dd, *J* = 8.9, 6.7 Hz, 1H), 6.97 (t, *J* = 6.9 Hz, 1H), 4.05 (s, 3H). ^13^C NMR (101 MHz, CDCl_3_) *δ* 160.0, 134.1, 133.5, 130.7, 128.7, 127.5, 127.4, 126.3, 125.8, 123.3, 118.6, 115.7, 52.2.

#### Butyl 1-phenylimidazo[1,5-*a*]pyridine-3-carboxylate(3c′)^19^

According to method A and purified by column chromatography with ethyl acetate/petroleum ether (v/v = 1 : 4) to obtain yellow solid (41.7 mg, 71%). ^1^H NMR (600 MHz, CDCl_3_) *δ* 9.40 (d, *J* = 7.2 Hz, 1H), 7.96 (d, *J* = 9.1 Hz, 1H), 7.90 (d, *J* = 7.3 Hz, 2H), 7.47 (t, *J* = 7.7 Hz, 2H), 7.35 (t, *J* = 7.4 Hz, 1H), 7.17–7.07 (m, 1H), 6.94 (dd, *J* = 10.0, 3.7 Hz, 1H), 4.48 (t, *J* = 7.0 Hz, 2H), 1.89–1.83 (m, 2H), 1.53–1.47 (m, 2H), 0.99 (dd, *J* = 8.6, 6.2 Hz, 3H). ^13^C NMR (151 MHz, CDCl_3_) *δ* 160.0, 134.1, 133.9, 130.8, 128.8, 127.6, 127.5, 126.8, 126.0, 123.2, 118.7, 115.6, 65.2, 30.9, 19.2, 13.9.

#### (1-Phenylimidazo[1,5-*a*]pyridin-3-yl)(*p*-tolyl)methanone (3d)^27^

According to method B and purified by column chromatography with ethyl acetate/petroleum ether (v/v = 1 : 4) to obtain yellow solid (45.6 mg, 73%). ^1^H NMR (600 MHz, CDCl_3_) *δ* 9.92 (d, *J* = 7.2 Hz), 8.47 (d, *J* = 8.2 Hz), 8.06 (d, *J* = 9.0 Hz), 7.98–7.95 (m), 7.51 (t, *J* = 7.7 Hz), 7.41–7.33 (m), 7.27–7.25 (m), 7.07–7.03 (m), 2.46 (s). ^13^C NMR (151 MHz, CDCl_3_) *δ* 182.0, 153.4, 142.8, 135.5, 134.4, 134.1, 134.0, 131.0, 128.8, 128.7, 127.5, 127.3, 124.9, 118.3, 116.2.

#### (2-Bromophenyl)(1-phenylimidazo[1,5-*a*]pyridin-3-yl)methanone (3e)

According to method B and purified by column chromatography with ethyl acetate/petroleum ether (v/v = 1 : 4) to obtain light yellow solid (58.1 mg, 77%). ^1^H NMR (600 MHz, CDCl_3_) *δ* 9.92 (dt, *J* = 7.1, 1.1 Hz), 8.08–8.06 (m), 7.87–7.80 (m), 7.69 (td, *J* = 7.9, 1.3 Hz), 7.47–7.41 (m), 7.37–7.32 (m), 7.14 (td, *J* = 6.9, 1.2 Hz). ^13^C NMR (151 MHz, CDCl_3_) *δ* 183.6, 140.3, 135.9, 133.6, 133.5, 133.2, 132.0, 131.0, 130.8, 128.7, 127.7, 127.4, 127.2, 126.6, 125.8, 120.6, 118.5, 116.9. HRMS (ESI) *m*/*z* [M + H^+^] calcd for C_20_H_13_BrN_2_O, 376.0211, found. 376.0218.

#### (3-Bromophenyl)(1-phenylimidazo[1,5-*a*]pyridin-3-yl)methanone (3f)

According to method B and purified by column chromatography with ethyl acetate/petroleum ether (v/v = 1 : 4) to obtain light yellow solid (59.6 mg, 79%). ^1^H NMR (600 MHz, CDCl_3_) *δ* 9.92 (dt, *J* = 7.2, 1.0 Hz), 8.67 (t, *J* = 1.8 Hz), 8.55–8.49 (m), 8.10 (dt, *J* = 9.0, 1.1 Hz), 7.99–7.93 (m), 7.70 (ddd, *J* = 7.9, 2.0, 1.0 Hz), 7.56–7.49 (m), 7.43–7.38 (m), 7.32 (ddd, *J* = 9.0, 6.6, 1.0 Hz), 7.11 (td, *J* = 7.0, 1.2 Hz). ^13^C NMR (151 MHz, CDCl_3_) *δ* 180.1, 139.9, 135.1, 134.8, 133.7, 133.7, 131.5, 129.6, 129.5, 128.9, 127.8, 127.4, 127.3, 125.5, 122.1, 118.5, 116.8. HRMS (ESI) *m*/*z* [M + H^+^] calcd for C_20_H_13_BrN_2_O, 376.0211, found. 376.0217.

#### (4-Bromophenyl)(1-phenylimidazo[1,5-*a*]pyridin-3-yl)methanone (3g)^28^

According to method B and purified by column chromatography with ethyl acetate/petroleum ether (v/v = 1 : 4) to obtain light yellow solid (57.3 mg, 76%). ^1^H NMR (600 MHz, CDCl_3_) *δ* 9.91 (dt, *J* = 7.1, 1.1 Hz), 8.47–8.43 (m), 8.08 (dt, *J* = 9.0, 1.2 Hz), 7.97–7.92 (m), 7.69–7.64 (m), 7.54–7.49 (m), 7.40 (ddd, *J* = 7.4, 4.1, 1.2 Hz), 7.30 (ddd, *J* = 9.0, 6.6, 1.0 Hz), 7.08 (td, *J* = 6.9, 1.2 Hz). ^13^C NMR (151 MHz, CDCl_3_) *δ* 180.6, 136.9, 134.9, 133.7, 132.5, 131.4, 131.2, 128.8, 127.7, 127.3, 127.2, 127.2, 125.4, 118.4, 116.6.

#### (4-Chlorophenyl)(1-phenylimidazo[1,5-*a*]pyridin-3-yl)methanone (3h)^27^

According to method B and purified by column chromatography with ethyl acetate/petroleum ether (v/v = 1 : 4) to obtain light yellow solid (53.8 mg, 81%). ^1^H NMR (600 MHz, CDCl_3_) *δ* 9.92 (d, *J* = 7.2 Hz), 8.56–8.51 (m), 8.09 (d, *J* = 9.0 Hz), 7.98–7.93 (m), 7.55–7.49 (m), 7.40 (d, *J* = 7.4 Hz), 7.31 (ddd, *J* = 8.9, 6.6, 0.8 Hz), 7.10 (dd, *J* = 6.8, 0.8 Hz). ^13^C NMR (151 MHz, CDCl_3_) *δ* 180.5, 138.4, 136.4, 134.9, 133.8, 133.8, 132.4, 131.4, 128.9, 128.3, 127.7, 127.3, 127.3, 125.4, 118.4, 116.6.

#### (4-Fluorophenyl)(1-phenylimidazo[1,5-*a*]pyridin-3-yl)methanone (3i)^27^

According to method B and purified by column chromatography with ethyl acetate/petroleum ether (v/v = 1 : 4) to obtain light yellow solid (49.9 mg, 79%). ^1^H NMR (600 MHz, CDCl_3_) *δ* 9.91 (d, *J* = 7.1 Hz), 8.65–8.59 (m), 8.08 (d, *J* = 9.0 Hz), 7.95 (d, *J* = 7.2 Hz), 7.52 (t, *J* = 7.7 Hz), 7.40 (d, *J* = 7.4 Hz), 7.29 (ddd, *J* = 9.0, 6.6, 0.6 Hz), 7.21 (t, *J* = 8.7 Hz), 7.10–7.04 (m). ^13^C NMR (151 MHz, CDCl_3_) *δ* 180.4, 166.1, 164.4, 134.7, 134.3, 134.3, 133.8, 133.5, 133.5, 131.2, 128.8, 127.7, 127.3, 127.3, 125.2, 118.4, 116.5, 115.1, 115.0.

#### (4-Iodophenyl)(1-phenylimidazo[1,5-*a*]pyridin-3-yl)methanone (3j)

According to method B and purified by column chromatography with ethyl acetate/petroleum ether (v/v = 1 : 4) to obtain light yellow solid (68.7 mg, 81%).^1^H NMR (600 MHz, CDCl_3_) *δ* 9.91 (d, *J* = 7.1 Hz), 8.28 (d, *J* = 8.4 Hz), 8.08 (d, *J* = 9.0 Hz), 7.94 (d, *J* = 7.3 Hz), 7.88 (d, *J* = 8.4 Hz), 7.51 (t, *J* = 7.7 Hz), 7.39 (t, *J* = 7.4 Hz), 7.30 (dd, *J* = 8.4, 7.1 Hz), 7.08 (t, *J* = 6.8 Hz). ^13^C NMR (151 MHz, CDCl_3_) *δ* 180.9, 137.4, 137.2, 134.9, 133.7, 132.4, 131.4, 128.8, 127.7, 127.3, 127.2, 125.4, 118.4, 116.6, 100.0. HRMS (ESI) *m*/*z* [M + H^+^] calcd for C_20_H_13_IN_2_O, 424.0073, found 424.0076.

#### (4-Methoxyphenyl)(1-phenylimidazo[1,5-*a*]pyridin-3-yl)methanone (3k)^27^

According to method B and purified by column chromatography with ethyl acetate/petroleum ether (v/v = 1 : 4) to obtain light yellow solid (47.9 mg, 73%). ^1^H NMR (600 MHz, CDCl_3_) *δ* 9.90 (d, *J* = 7.1 Hz), 8.67–8.61 (m), 8.05 (d, *J* = 9.0 Hz), 7.97 (dd, *J* = 8.1, 0.9 Hz), 7.51 (t, *J* = 7.7 Hz), 7.38 (t, *J* = 7.4 Hz), 7.26–7.22 (m), 7.05–7.00 (m), 3.91 (s). ^13^C NMR (151 MHz, CDCl_3_) *δ* 180.8, 162.9, 134.1, 134.1, 134.0, 133.2, 130.8, 130.8, 128.8, 127.4, 127.2, 124.7, 118.3, 116.1, 113.3, 55.4.

#### (1-Phenylimidazo[1,5-*a*]pyridin-3-yl)(4-(trifluorome-thyl)phenyl)methanone (3l)^27^

According to method B and purified by column chromatography with ethyl acetate/petroleum ether (v/v = 1 : 4) to obtain yellow solid (52.0 mg, 71%). ^1^H NMR (600 MHz, CDCl_3_) *δ* 9.95 (dt, *J* = 7.2, 1.2 Hz), 8.66–8.60 (m), 8.11 (dt, *J* = 9.1, 1.3 Hz), 7.99–7.92 (m), 7.85–7.75 (m), 7.56–7.50 (m), 7.49–7.36 (m), 7.35 (ddd, *J* = 9.1, 6.6, 1.2 Hz), 7.16–7.10 (m). ^13^C (151 MHz, CDCl_3_) *δ* 180.6, 141.2, 135.4, 133.7, 133.6, 133.2, 133.0, 131.7, 131.1, 128.9, 127.9, 127.4, 127.3, 125.7, 124.9, 124.9, 124.9, 124.9, 118.5, 116.9.

#### 4-(1-Phenylimidazo[1,5-*a*]pyridine-3-carbonyl)benzonitrile(3m)^27^

According to method B and purified by column chromatography with ethyl acetate/petroleum ether (v/v = 1 : 4) to obtain yellow solid (42.6 mg, 66%). ^1^H NMR (600 MHz, CDCl_3_) *δ* 9.93 (dd, *J* = 7.1, 0.8 Hz), 8.67–8.53 (m), 8.11 (d, *J* = 9.0 Hz), 7.93 (dd, *J* = 8.1, 0.9 Hz), 7.83–7.77 (m), 7.53 (dd, *J* = 10.7, 4.8 Hz), 7.45–7.38 (m), 7.37 (ddd, *J* = 8.8, 6.6, 0.7 Hz), 7.19–7.10 (m). ^13^C NMR (151 MHz, CDCl_3_) *δ* 179.6, 141.7, 135.6, 133.6, 133.4, 131.8, 131.7, 131.2, 128.9, 128.0, 127.4, 127.3, 126.0, 118.6, 118.4, 117.1, 114.9.

#### (3-Nitrophenyl)(1-phenylimidazo[1,5-*a*]pyridin-3-yl)methanone (3n)

According to method B and purified by column chromatography with ethyl acetate/petroleum ether (v/v = 1 : 4) to obtain yellow solid (50.1 mg, 73%). ^1^H NMR (600 MHz, CDCl_3_) *δ* 9.94 (dt, *J* = 7.0, 0.9 Hz), 9.55–9.50 (m), 8.89–8.84 (m), 8.42 (ddd, *J* = 8.2, 2.3, 1.0 Hz), 8.13 (dt, *J* = 9.0, 1.1 Hz), 7.96 (dd, *J* = 8.3, 1.2 Hz), 7.71 (t, *J* = 7.9 Hz), 7.53 (t, *J* = 7.8 Hz), 7.44–7.35 (m), 7.16 (td, *J* = 6.9, 1.2 Hz). ^13^C NMR (151 MHz, CDCl_3_) *δ* 178.6, 147.9, 139.4, 136.4, 135.6, 133.4, 133.4, 131.8, 129.0, 129.0, 128.0, 127.4, 127.2, 126.2, 126.2, 126.0, 118.6, 117.2. HRMS (ESI) *m*/*z* [M + H^+^] calcd for C_20_H_13_N_3_O_3_ 343.0957, found 343.0952.

#### (2,4-Dichlorophenyl)(1-phenylimidazo[1,5-*a*]pyridin-3-yl)methanone (3p)^27^

According to method B and purified by column chromatography with ethyl acetate/petroleum ether (v/v = 1 : 4) to obtain yellow solid (53.6 mg, 73%). ^1^H NMR (600 MHz, CDCl_3_) *δ* 9.90 (dt, *J* = 7.1, 1.1 Hz), 8.08 (dt, *J* = 9.0, 1.2 Hz), 7.87–7.83 (m), 7.69 (d, *J* = 8.2 Hz), 7.52 (d, *J* = 1.9 Hz), 7.49–7.43 (m), 7.40–7.34 (m), 7.16 (td, *J* = 6.9, 1.2 Hz). ^13^C NMR (151 MHz, CDCl_3_) *δ* 181.5, 136.8, 136.3, 136.0, 133.6, 133.4, 133.2, 132.1, 131.8, 130.0, 128.8, 127.9, 127.4, 127.2, 126.5, 126.0, 118.5, 117.1.

#### (2,6-Dichlorophenyl)(1-phenylimidazo[1,5-*a*]pyridin-3-yl)methanone (3q)

According to method B and purified by column chromatography with ethyl acetate/petroleum ether (v/v = 1 : 4) to obtain yellow solid (48.4 mg, 66%).^1^H NMR (600 MHz, CDCl_3_) *δ* 9.89 (d, *J* = 7.0 Hz), 8.07 (d, *J* = 9.0 Hz), 7.82 (d, *J* = 8.1 Hz), 7.44 (t, *J* = 7.6 Hz), 7.41–7.31 (m), 7.17 (t, *J* = 6.9 Hz). ^13^C NMR (151 MHz, CDCl_3_) *δ* 180.7, 138.2, 136.5, 133.5, 132.5, 132.4, 131.1, 130.3, 128.7, 127.9, 127.8, 127.5, 127.1, 126.1, 118.6, 117.2. HRMS (ESI) *m*/*z* [M + H^+^] calcd for C_20_H_12_Cl_2_N_2_O 366.0327, found 366.0330.

#### (2,4-Difluorophenyl)(1-phenylimidazo[1,5-*a*]pyridin-3-yl)methanone (3r)

According to method B and purified by column chromatography with ethyl acetate/petroleum ether (v/v = 1 : 4) to obtain light yellow solid (47.4 mg, 71%). ^1^H NMR (600 MHz, CDCl_3_) *δ* 9.87 (d, *J* = 7.1 Hz), 8.07 (d, *J* = 9.0 Hz), 7.98 (td, *J* = 8.2, 6.7 Hz), 7.91–7.85 (m), 7.48 (t, *J* = 7.7 Hz), 7.40–7.30 (m), 7.13 (td, *J* = 7.0, 1.0 Hz), 7.03–6.98 (m), 6.98–6.91 (m). ^13^C NMR (151 MHz, CDCl_3_) *δ* 179.5, 165.3, 163.6, 162.2, 160.5, 135.6, 133.8, 133.6, 133.3, 133.3, 131.9, 128.8, 127.8, 127.3, 127.1, 125.7, 124.0, 118.5, 116.9, 111.1, 110.9, 104.8, 104.6, 104.4. HRMS (ESI) *m*/*z* [M + H^+^] calcd for C_20_H_12_F_2_N_2_O 334.0918, found 334.0915.

#### (1-Phenylimidazo[1,5-*a*]pyridin-3-yl)(2,4,5-trifluorophenyl) methanone (3s)

According to method B and purified by column chromatography with ethyl acetate/petroleum ether (v/v = 1 : 4) to obtain light yellow solid (45.8 mg, 65%). ^1^H NMR (600 MHz, CDCl_3_) *δ* 9.85 (d, *J* = 7.1 Hz), 8.09 (d, *J* = 9.0 Hz), 7.91–7.80 (m), 7.50 (t, *J* = 7.7 Hz), 7.42–7.34 (m), 7.16 (td, *J* = 7.0, 0.8 Hz), 7.07 (td, *J* = 9.7, 6.3 Hz). ^13^C NMR (151 MHz, CDCl_3_) *δ* 177.8, 157.21, 157.1, 155.5, 155.4, 152.7, 152.6, 150.9, 150.9, 147.1, 147.0, 145.4, 145.3, 136.0, 133.5, 133.4, 132.2, 128.9, 128.0, 127.3, 127.1, 126.1, 123.7, 123.6, 119.9, 119.8, 118.6, 117.2, 106.5, 106.3, 106.3, 106.2. HRMS (ESI) *m*/*z* [M + H^+^] calcd for C_20_H_11_F_3_N_2_O, 352.0823, found 352.0827.

#### (1-Phenylimidazo[1,5-*a*]pyridin-3-yl)(pyridin-3-yl)methanone (3t)

According to method B and purified by column chromatography with ethyl acetate/petroleum ether (v/v = 1 : 2) to obtain light yellow solid (37.6 mg, 63%). ^1^H NMR (600 MHz, CDCl3) *δ* 10.05–9.94 (m), 9.93 (dd, *J* = 7.2, 0.9 Hz), 9.78–9.69 (m), 8.86–8.68 (m), 8.12–8.01 (m), 7.99–7.77 (m), 7.49 (ddd, *J* = 10.6, 7.9, 6.3 Hz), 7.40 (d, *J* = 7.4 Hz), 7.36–7.30 (m), 7.13 (d, *J* = 6.9 Hz). ^13^C NMR (151 MHz, CDCl_3_) *δ* 179.8, 152.0, 151.9, 138.2, 135.4, 133.8, 133.6, 133.5, 131.7, 128.9, 127.8, 127.3, 127.2, 125.8, 123.0, 118.5, 117.0. HRMS (ESI) *m*/*z* [M + H^+^] calcd for C_19_H_13_N_3_O, 299.1059, found 299.1064.

## Author contributions

Xinyu Song, Shujun Qin, Wenya Dong, Pingjing Xue and Jiayi Wu contributed equally to the experimental work, Haibo Wang contributed to the modification of the manuscript. All authors reviewed and approved the submitted manuscript.

## Conflicts of interest

There are no conflicts to declare.

## Supplementary Material

RA-015-D5RA07797D-s001

## Data Availability

The data that support this study are available in the supplementary information (SI). Supplementary information: other experimental procedures, characterization data, and copies of NMR spectra for the compounds. See DOI: https://doi.org/10.1039/d5ra07797d.
