# Supplementary material for: Controllable site-selective C–C bond cleavage for the divergent synthesis of imidazo[1,5-a]pyridine derivatives
Source: RSC Adv. 2025 Dec 10;15(57):49189–96. doi: 10.1039/d5ra07797d (PMC12690639; doi:10.1039/d5ra07797d)

Supplementary Information (SI) for RSC Advances.
This journal is © The Royal Society of Chemistry 2025

Controllable Site-selective C-C Bond Cleavage for the Divergent Synthesis of Imidazo[1,5-a]pyridine Derivatives

Qiang Wang*, Xinyu Song, Shujun Qin, Wenya Dong, Pingjing Xue, Jiayi Wu, and Haibo Wang

College of biomedical and Health Sciences, Anhui Science and Technology University, Fengyang 233100, PR China.

List of Contents

1. **^{1}H NMR and ^{13}C NMR spectrogram S2-S20**
2. **Procedure of detection of crucial intermediates by LCMS S21-S22**

1. **^{1}H NMR and ^{13}C NMR spectrogram**

phenyl(1-phenylimidazo[1,5-a]pyridin-3-yl)methanone ***(3a)***

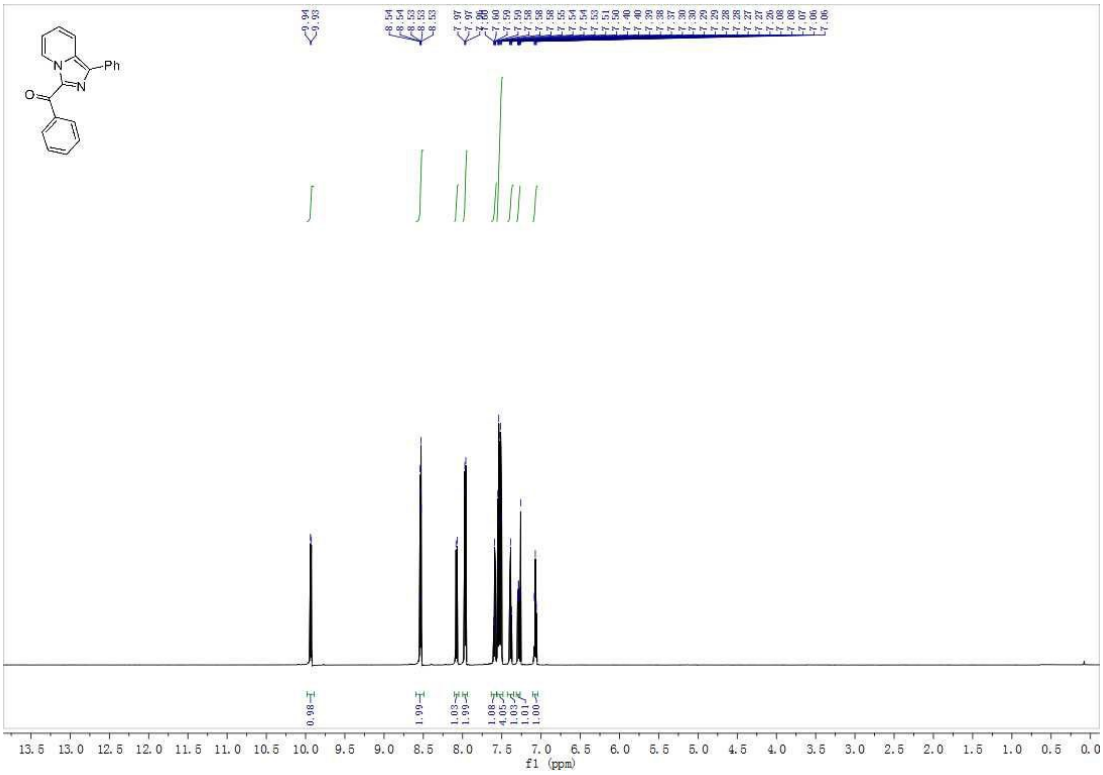

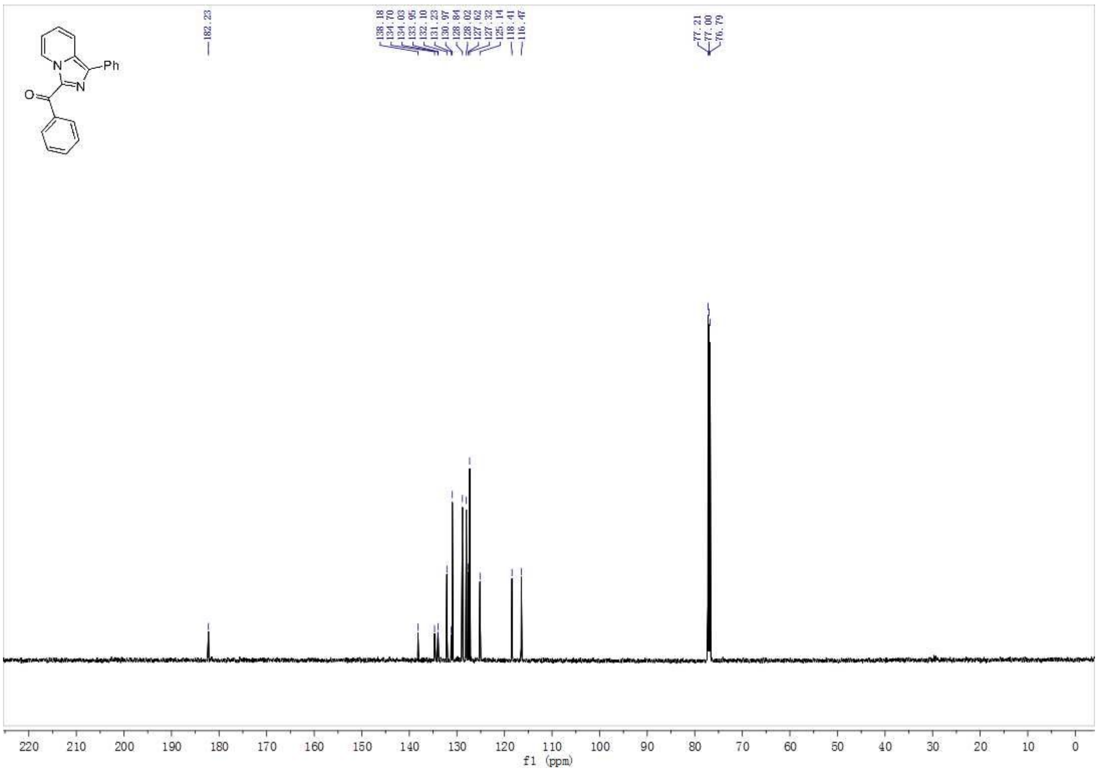

*(1-phenylimidazo[1,5-a]pyridin-3-yl)(o-tolyl)methanone (**3b**)*

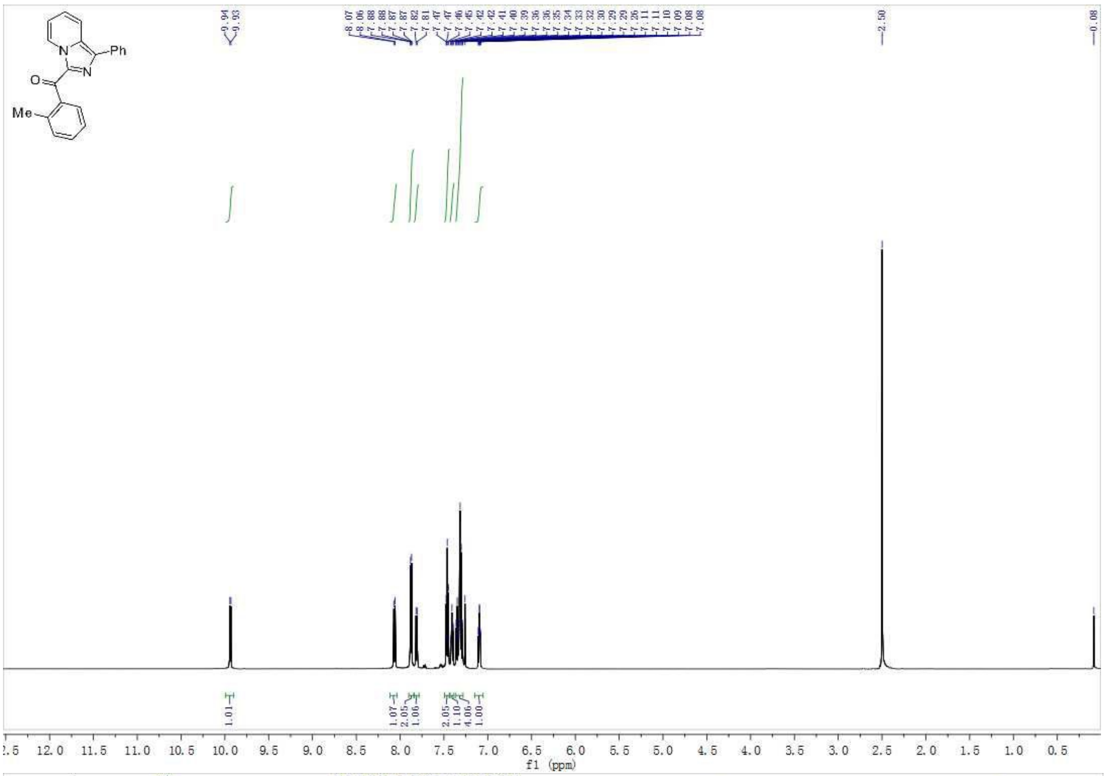

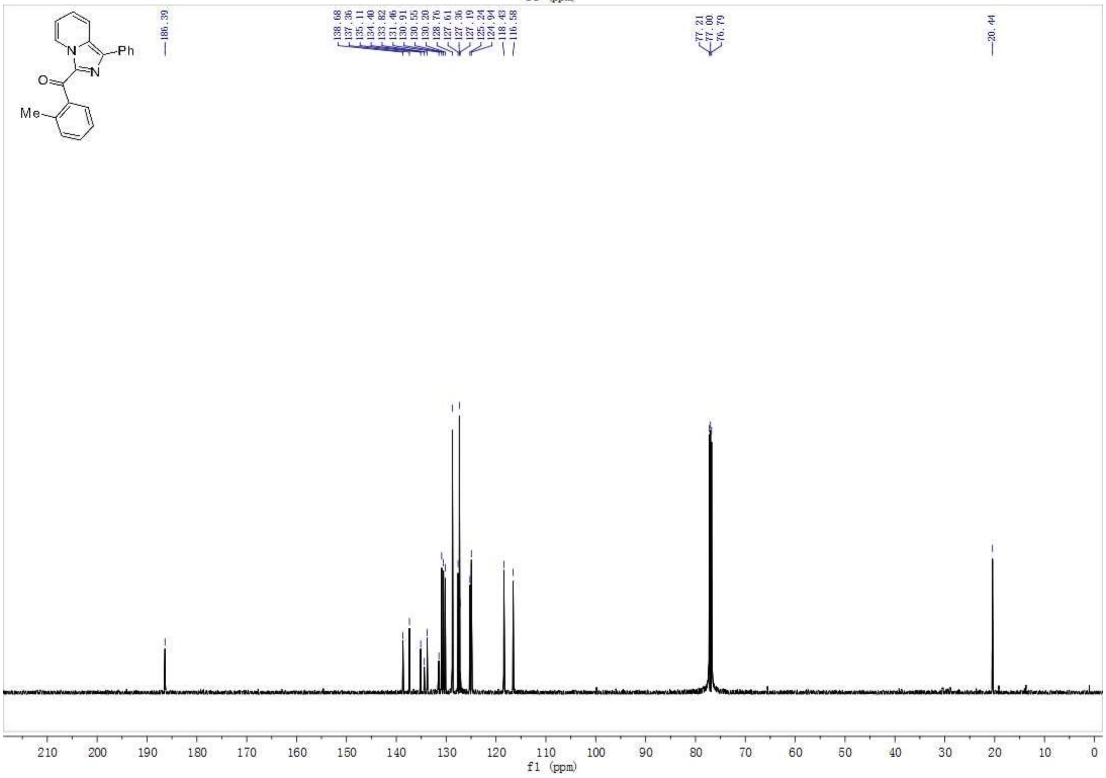

*(1- phenylimidazo[1,5-a]pyridin-3-yl)(m-tolyl)methanone (**3c**)*

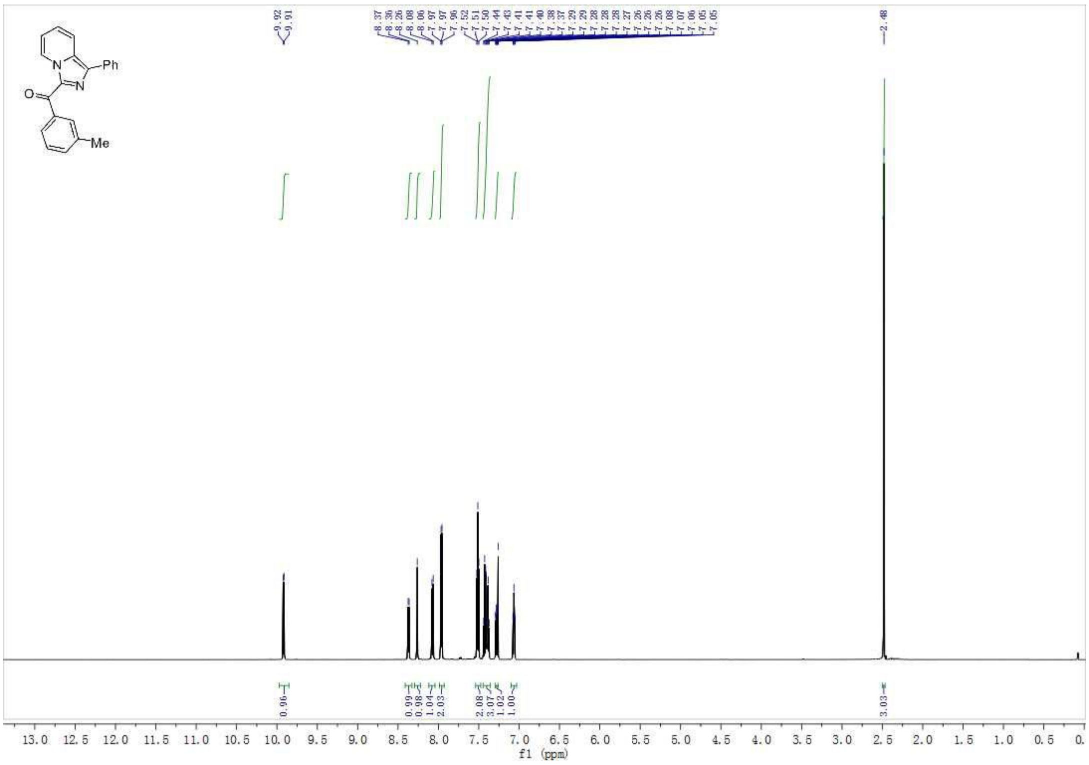

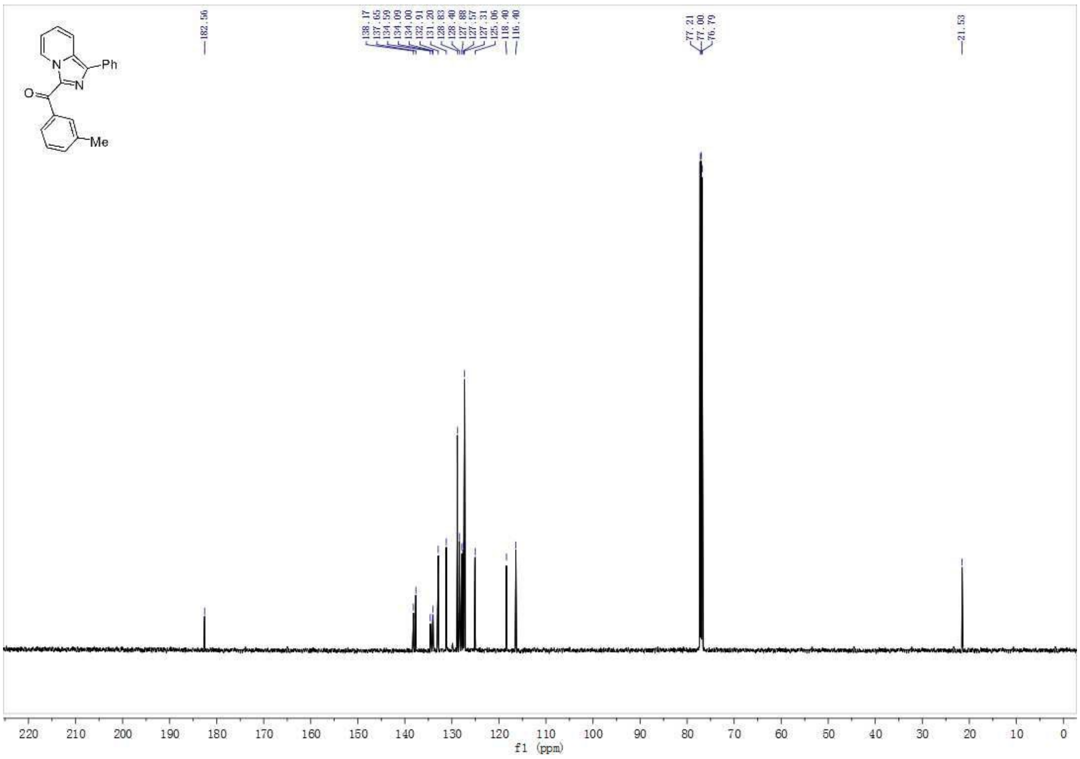

(1- phenylimidazo[1,5-a]pyridin-3-yl)(p-tolyl)methanone (3d)

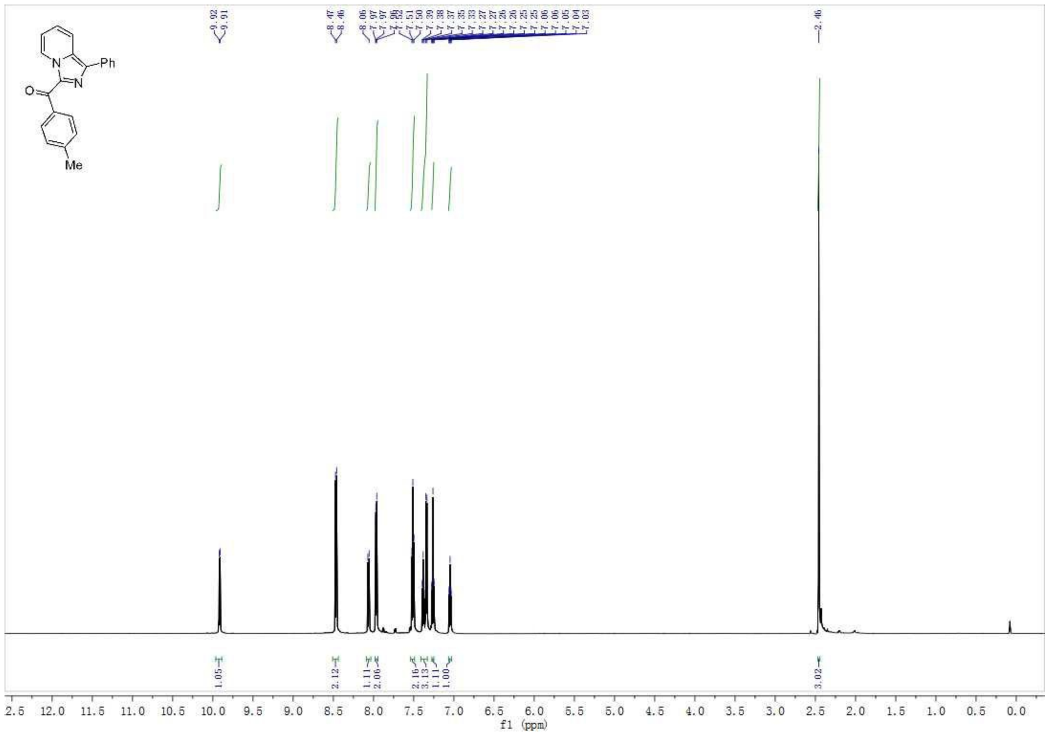

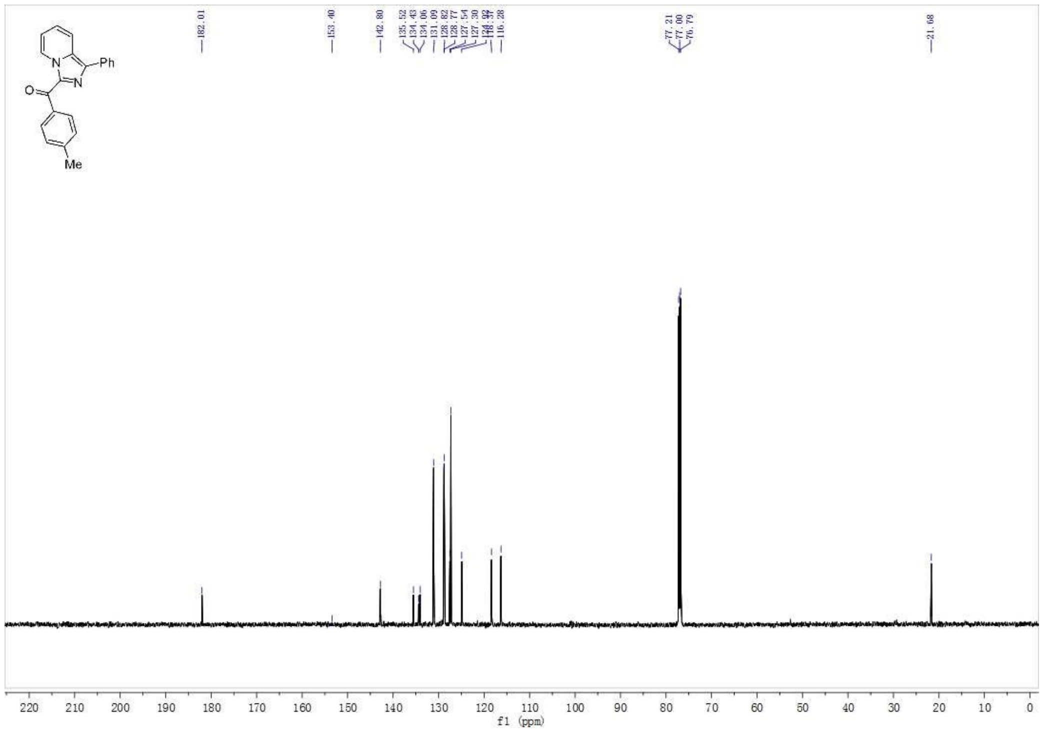

*(2- bromophenyl)(1-phenylimidazo[1,5-a]pyridin-3-yl)methanone (**3e**)*

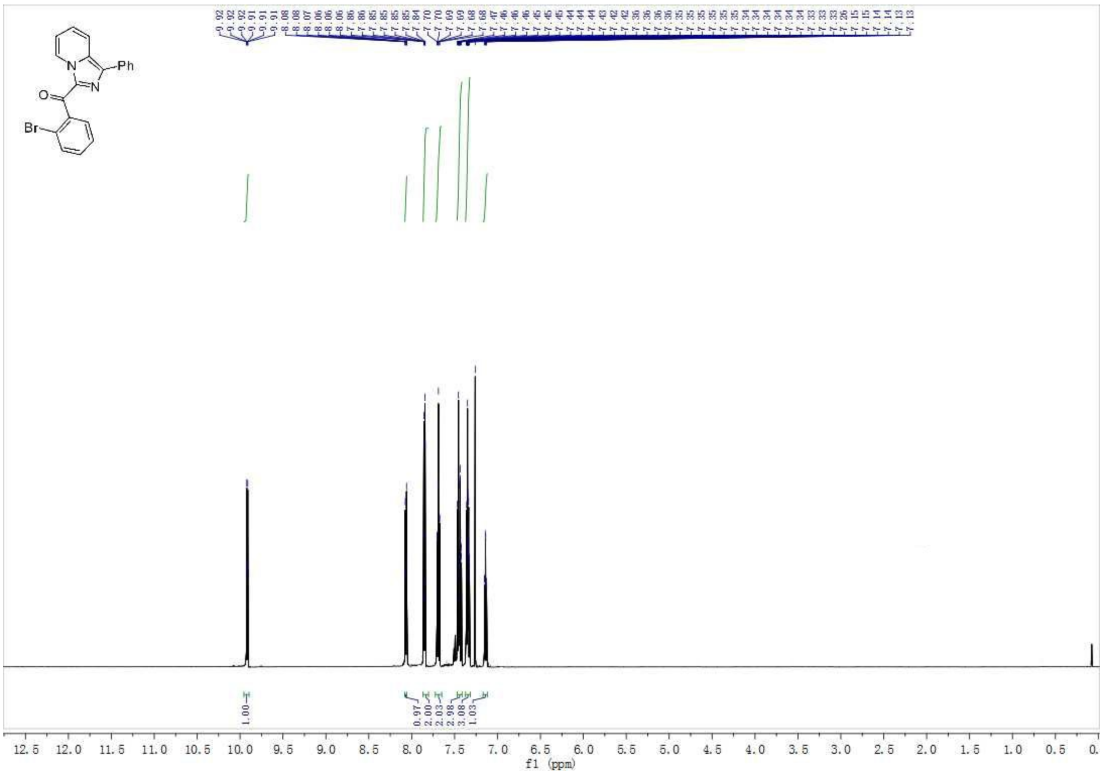

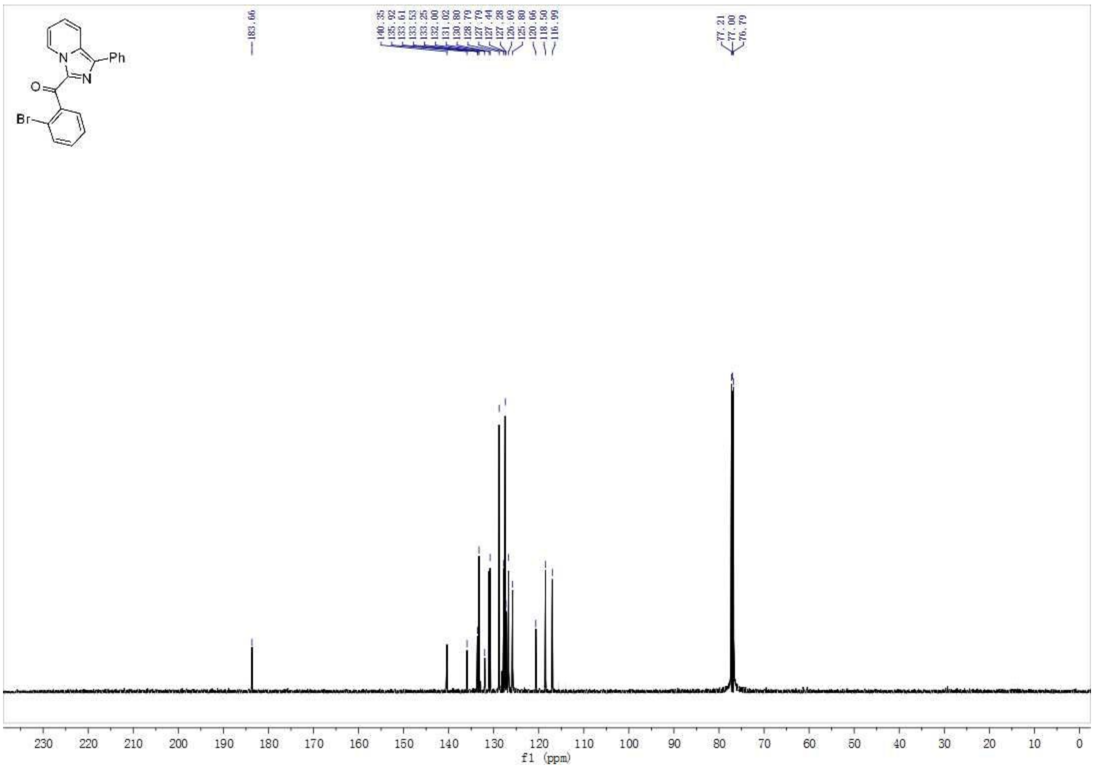

*(3- bromophenyl)(1-phenylimidazo[1,5-a]pyridin-3-yl)methanone (**3f**)*

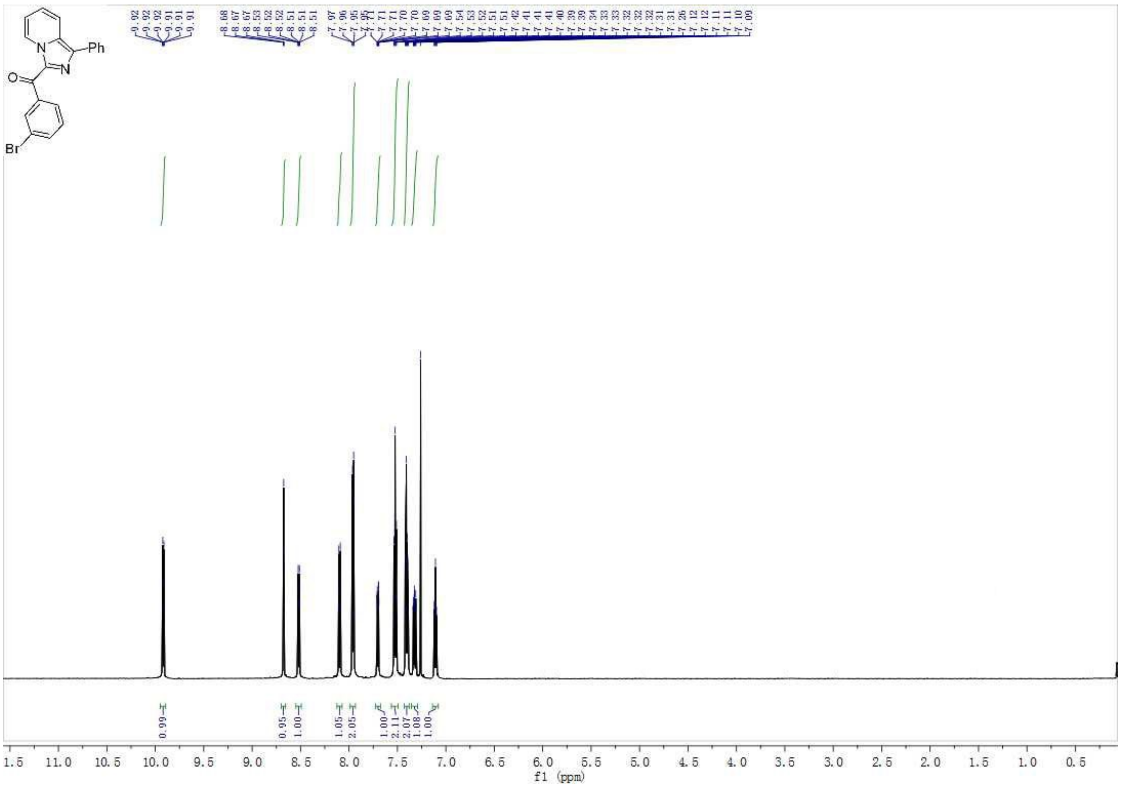

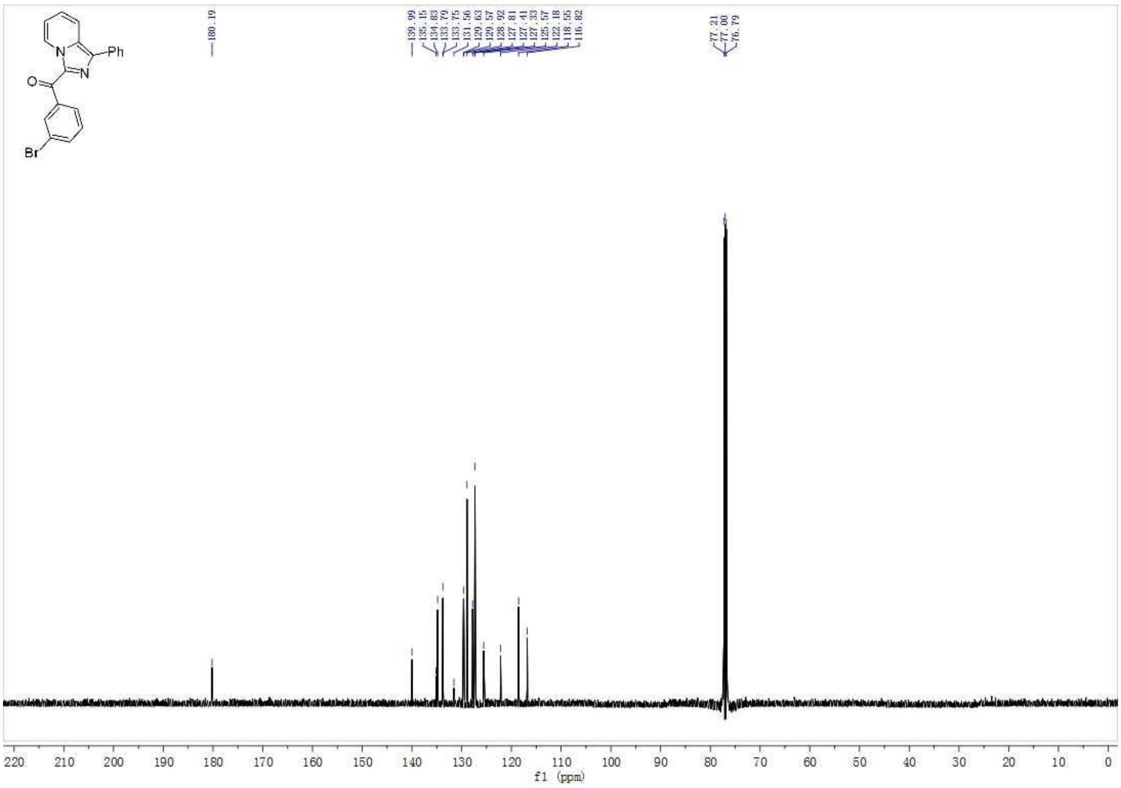

*(4-bromophenyl)(1-phenylimidazo[1,5-a]pyridin-3-yl)methanone (**3g**)*

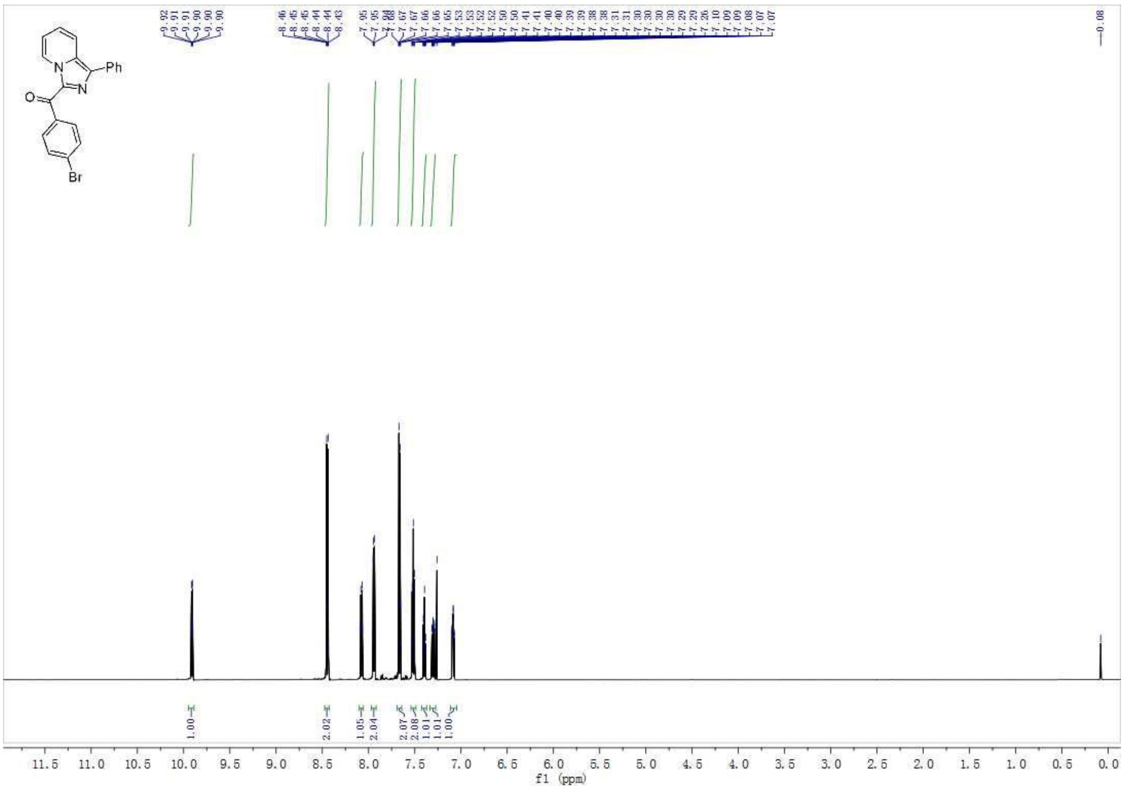

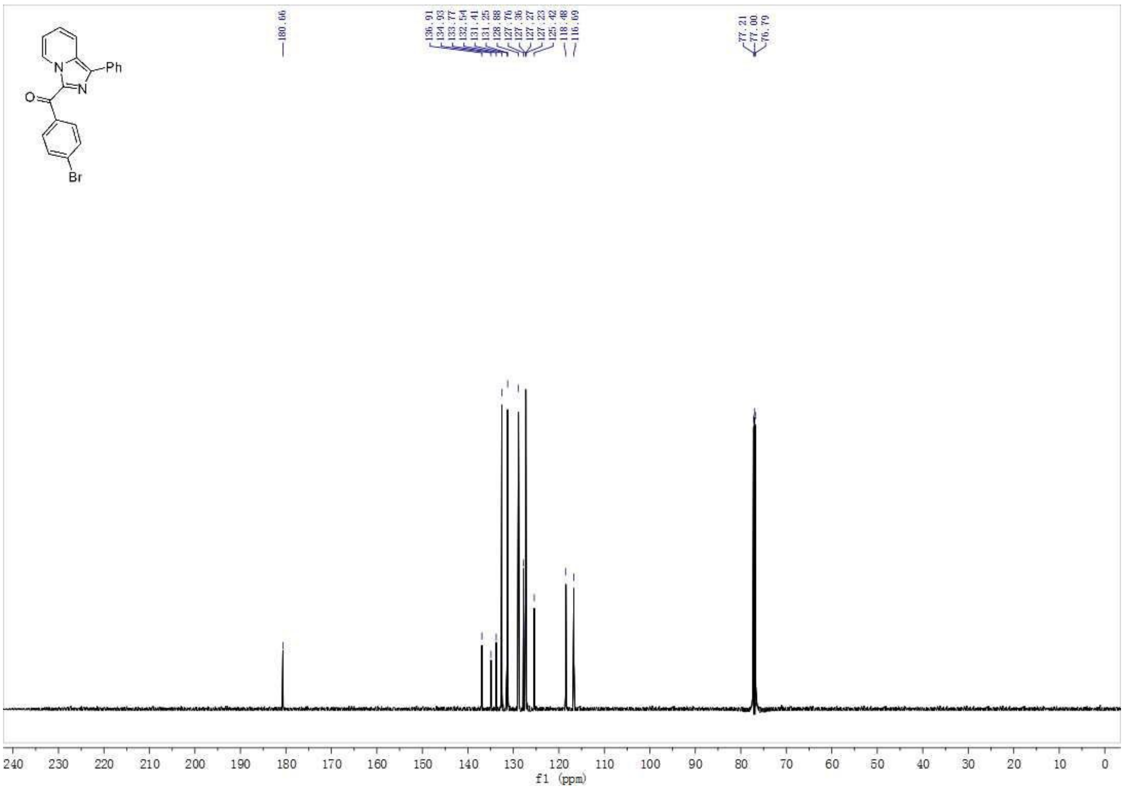

*(4-chlorophenyl)(1-phenylimidazo[1,5-a]pyridin-3-yl)methanone (**3h**)*

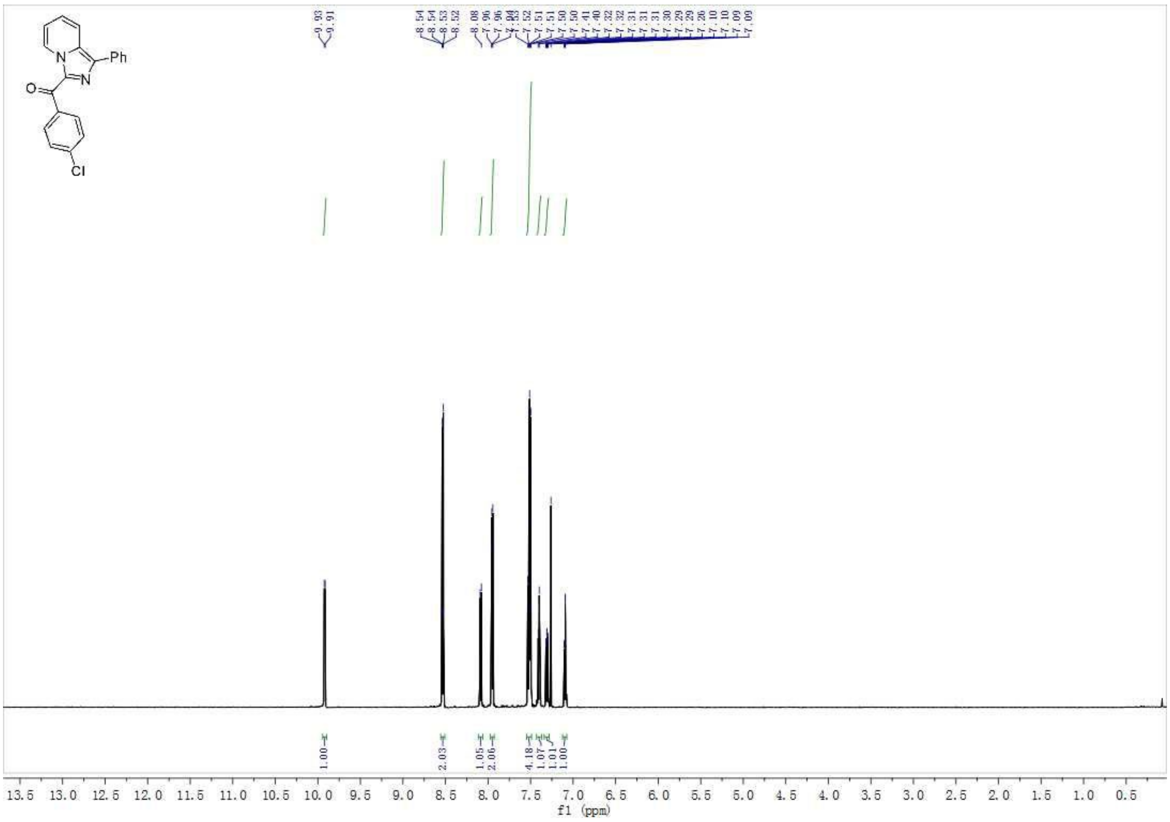

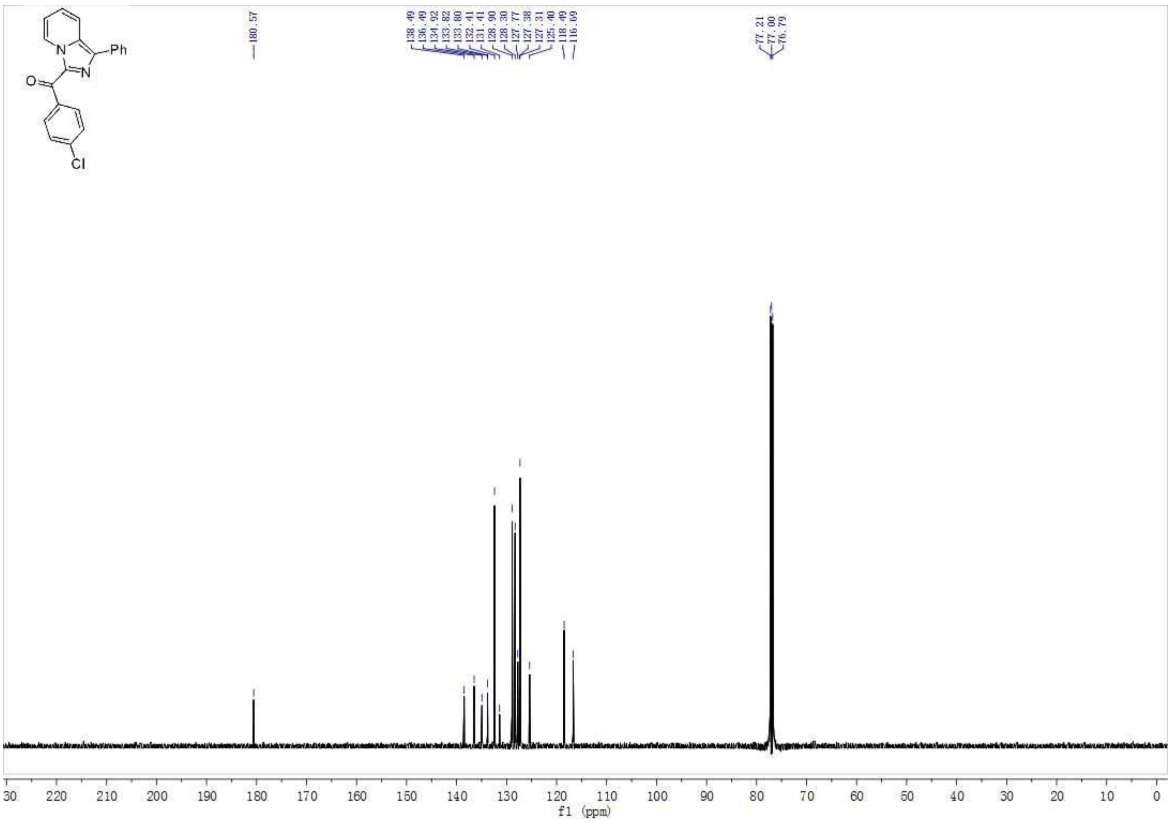

*(4-fluorophenyl)(1-phenylimidazo[1,5-a]pyridin-3-yl)methanone (**3i**)*

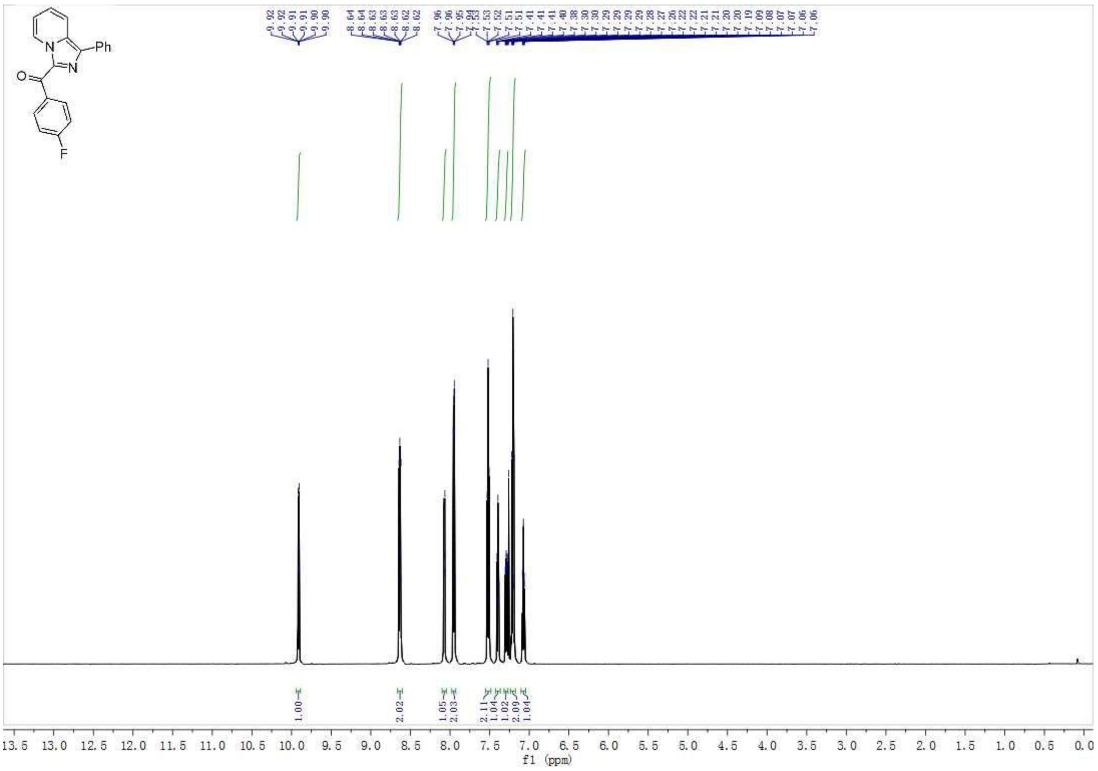

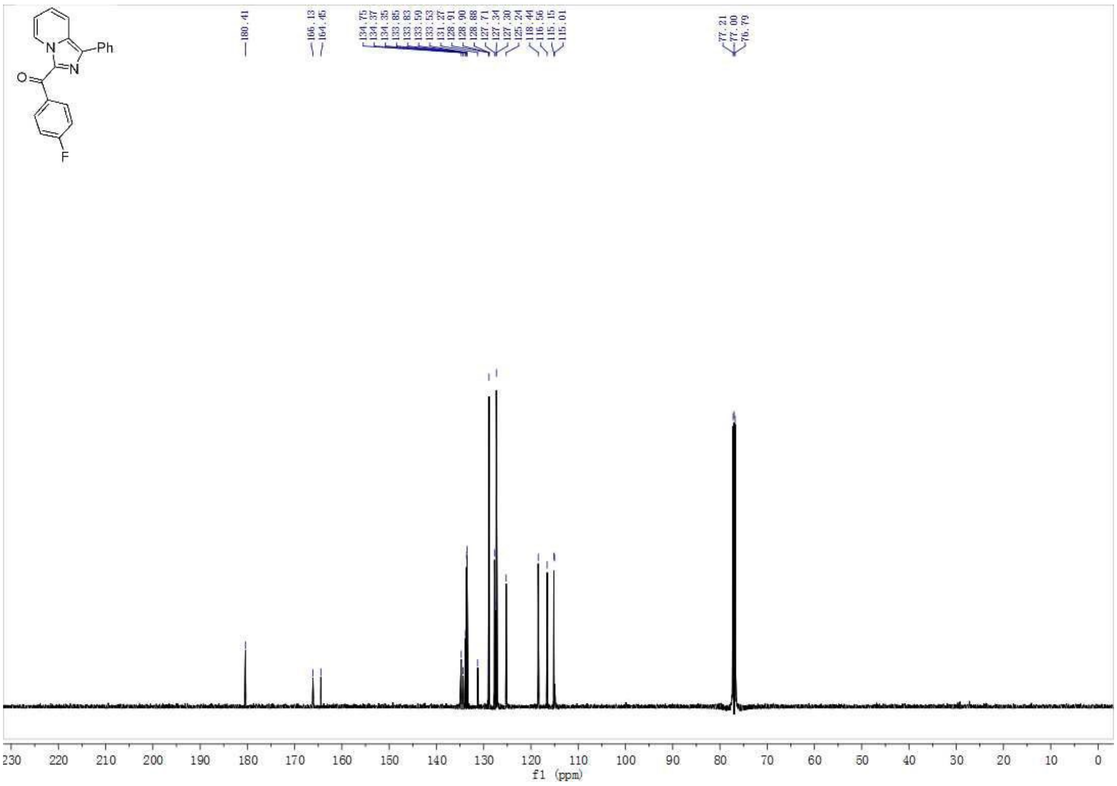

.

.

*(4-iodophenyl)(1-phenylimidazo[1,5-a]pyridin-3-yl)methanone (**3j**)*

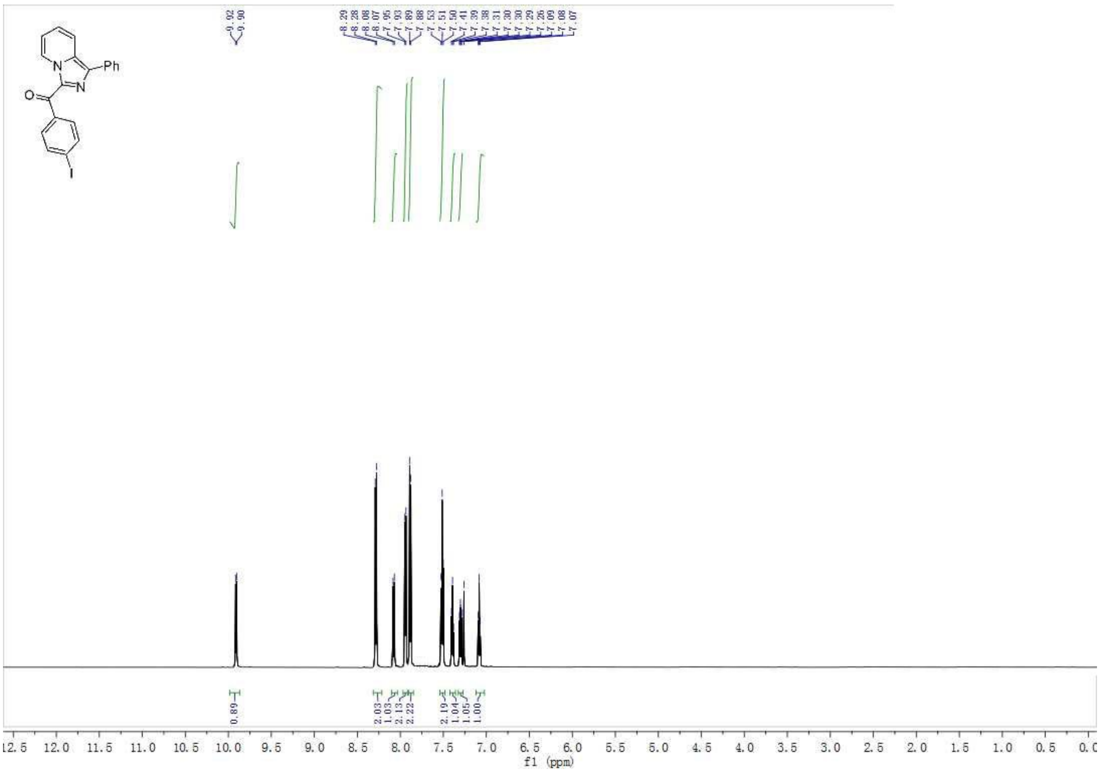

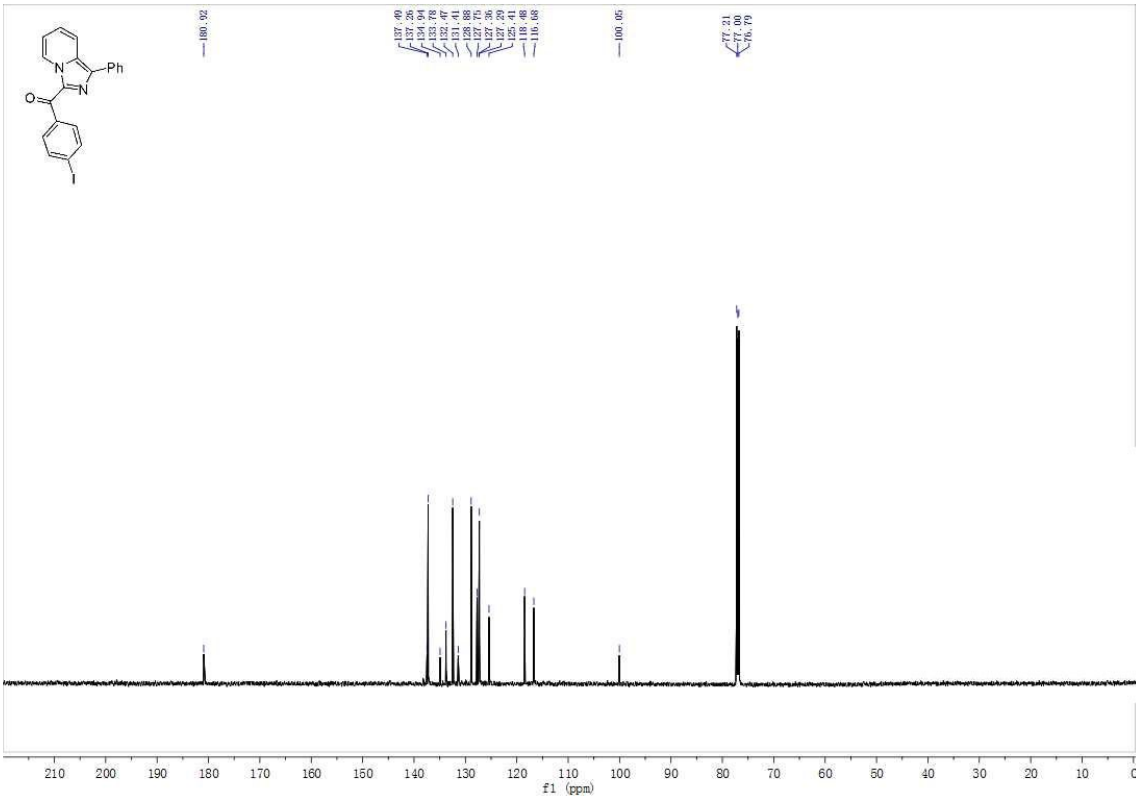

*(4-methoxyphenyl)(1-phenylimidazo[1,5-a]pyridin-3-yl)methanone (**3k**)*

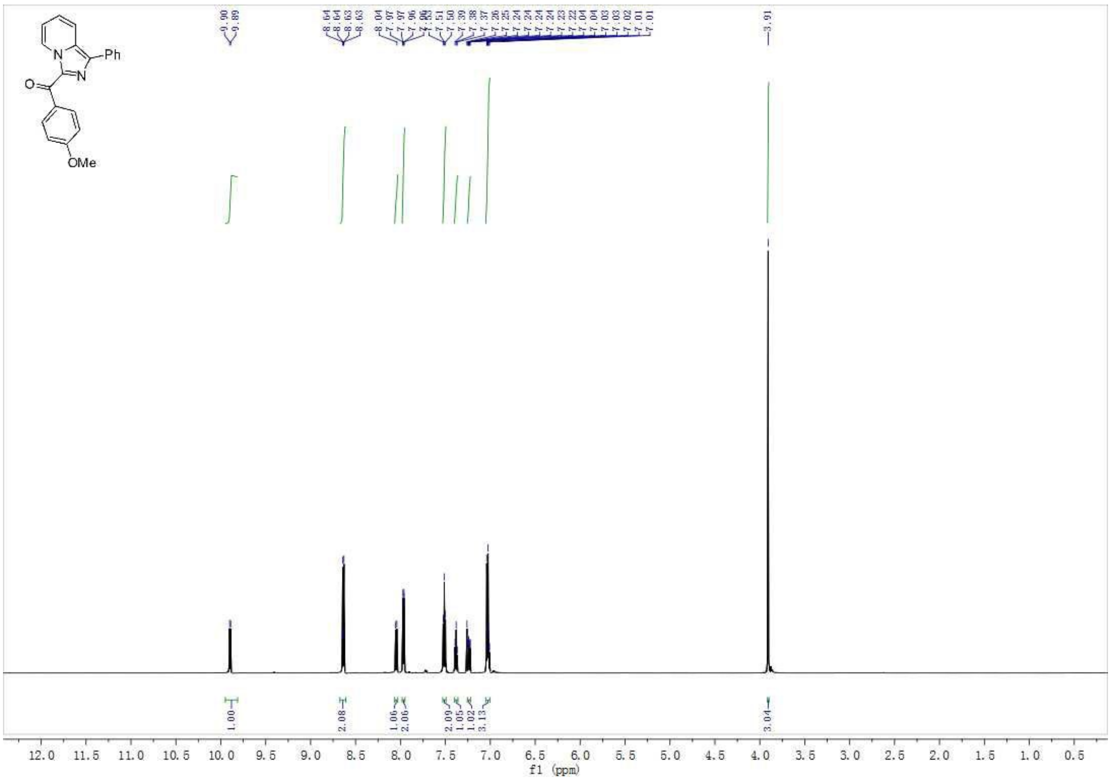

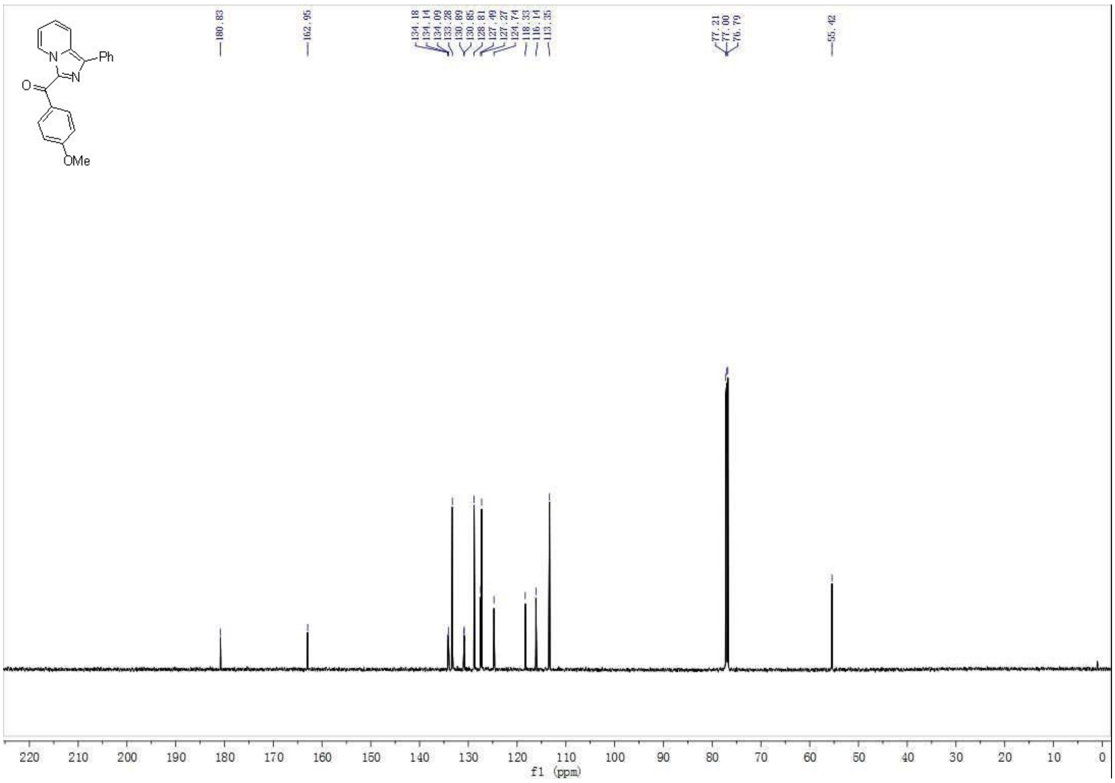

*(1-phenylimidazo[1,5-a]pyridin-3-yl)(4-(trifluorome-thyl)phenyl)methanone (**3l**)*

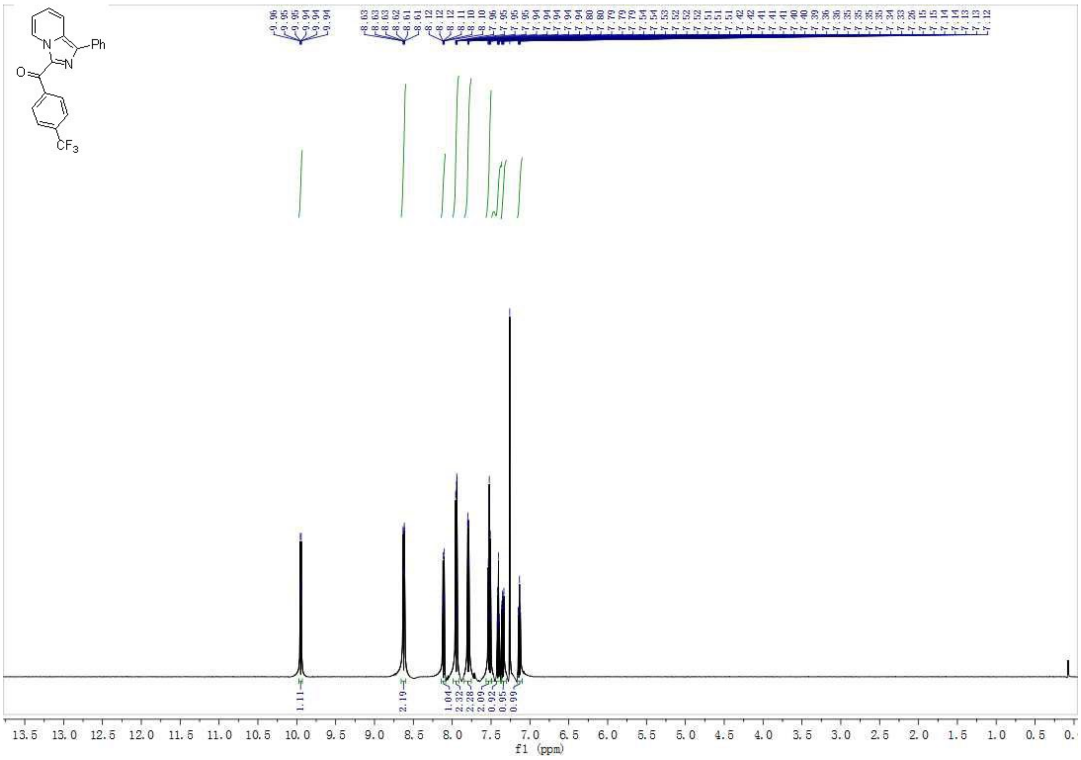

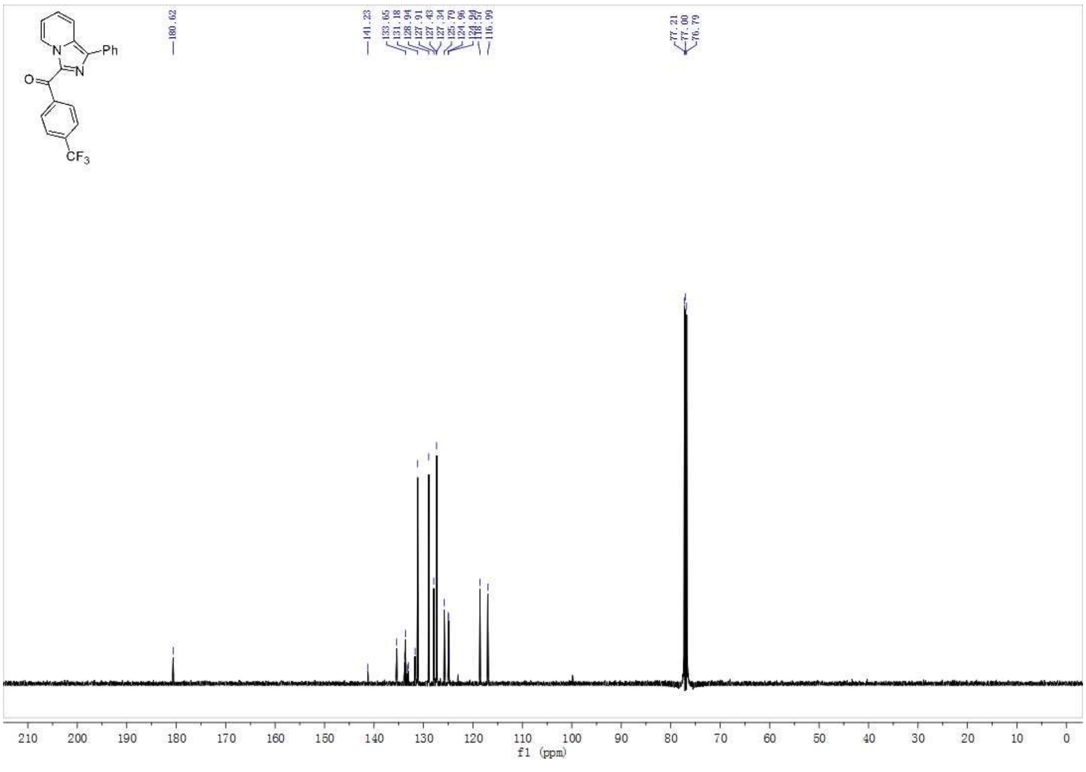

*4-(1-phenylimidazo[1,5-a]pyridine-3-carbonyl)benzonitrile (**3m**)*

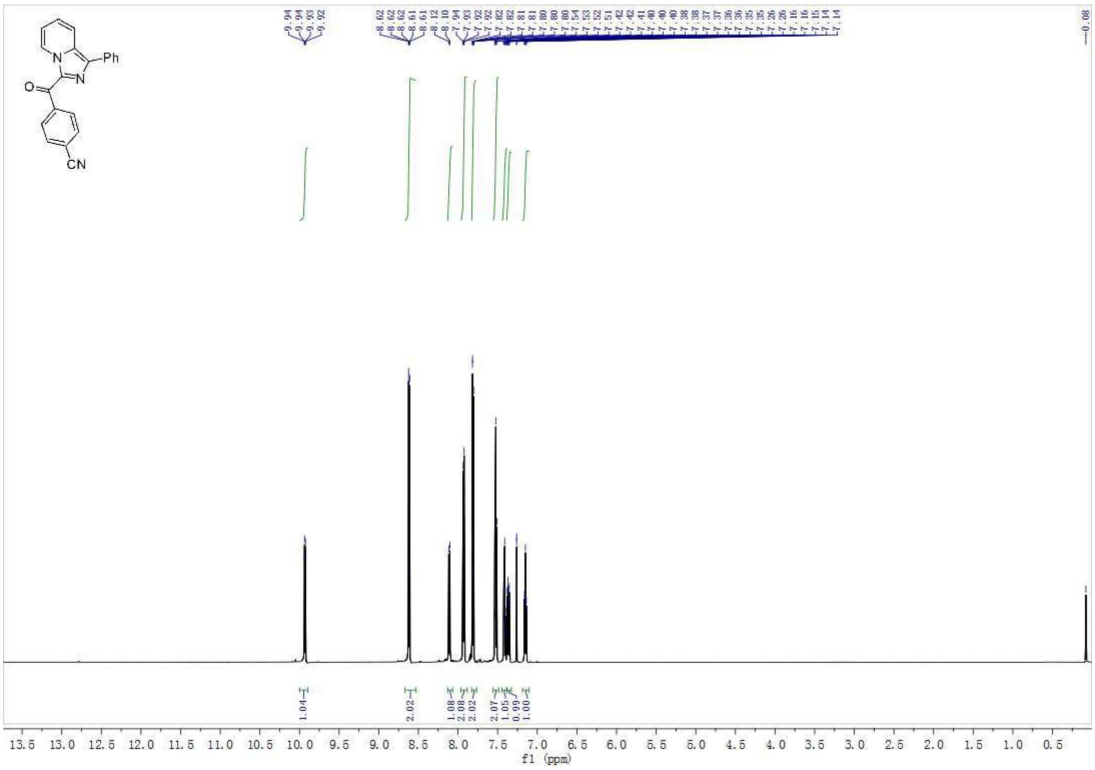

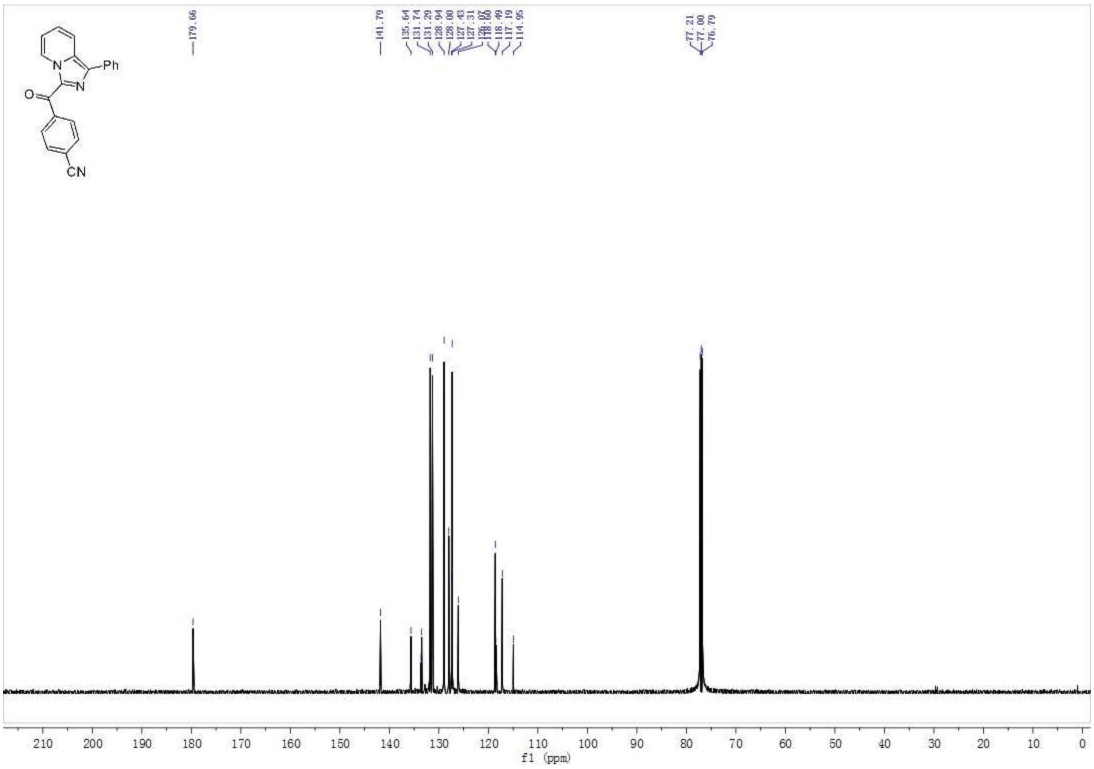

*(3-nitrophenyl)(1-phenylimidazo[1,5-a]pyridin-3-yl)methanone (**3n**)*

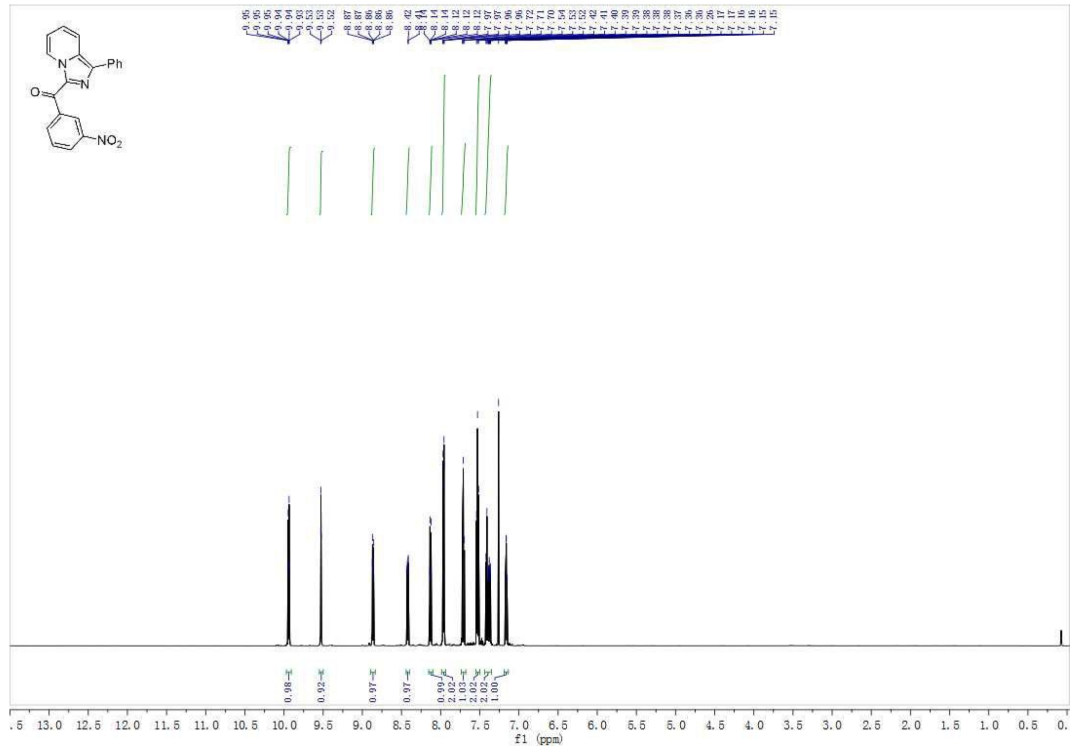

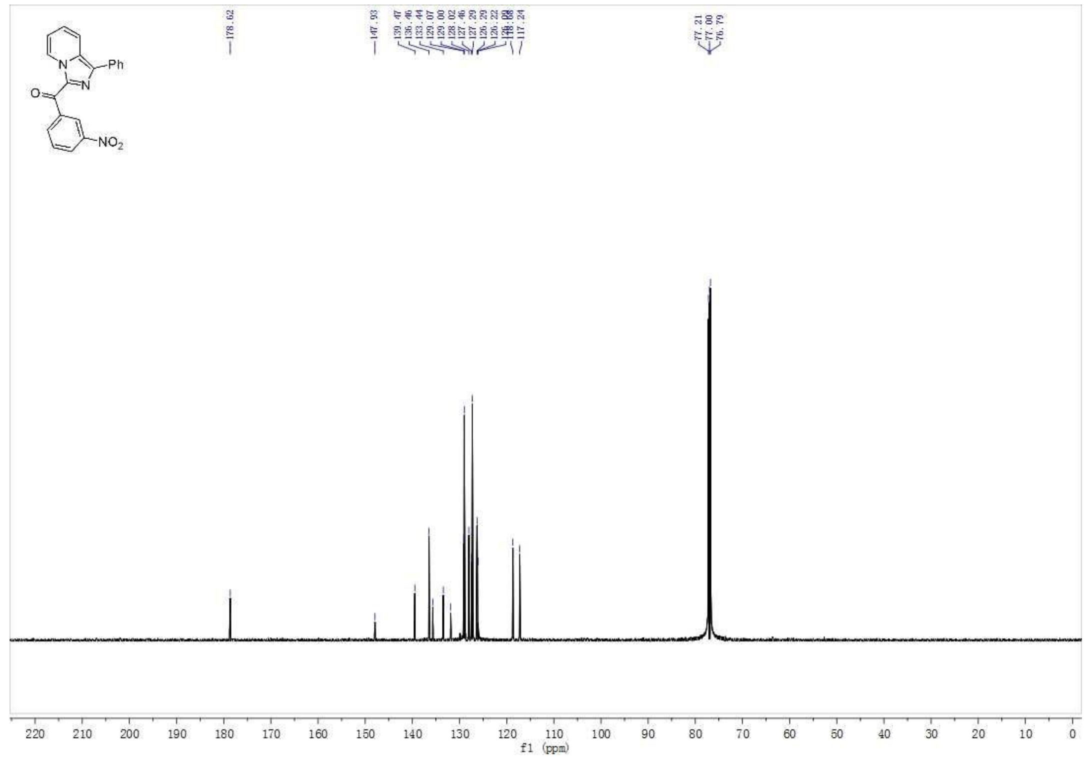

*(2,4-dichlorophenyl)(1-phenylimidazo[1,5-a]pyridin-3-yl)methanone (**3p**)*

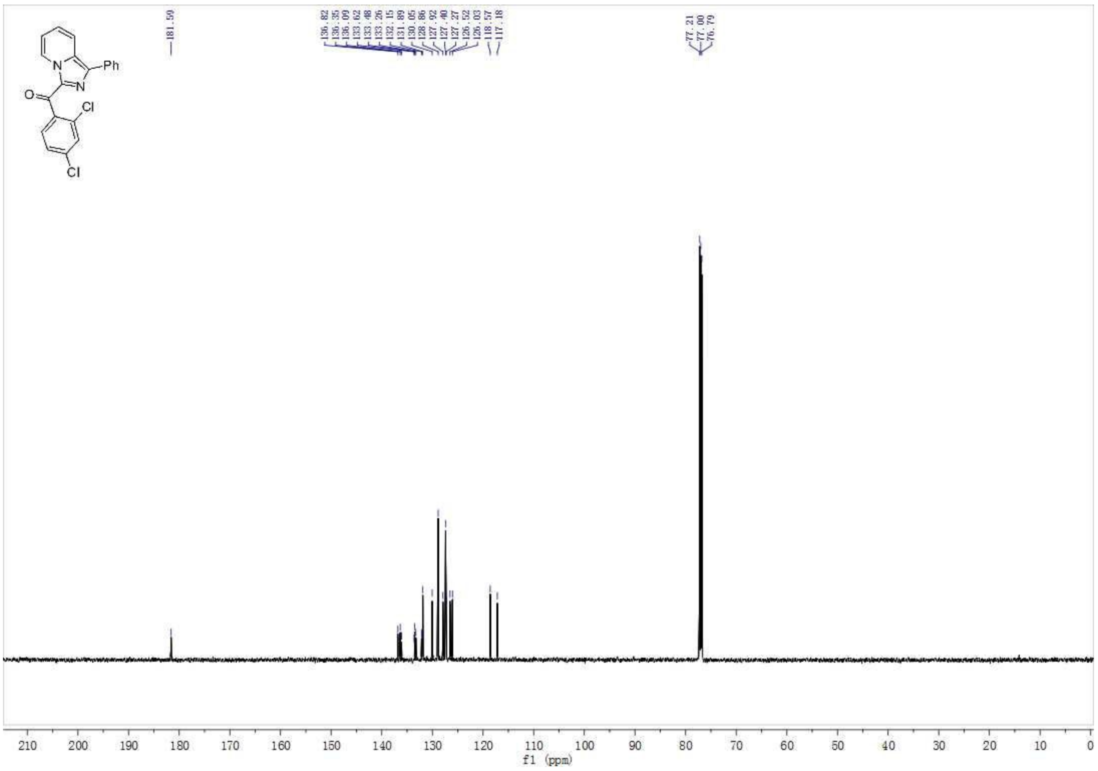

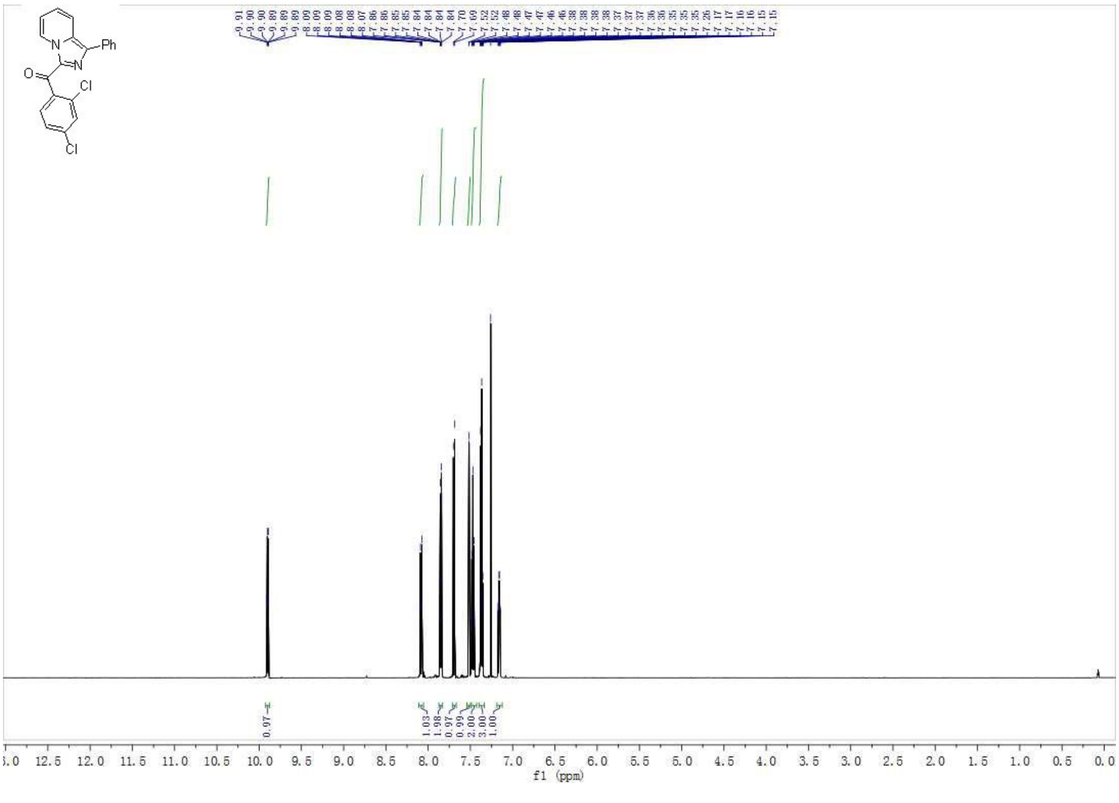

*(2,6-dichlorophenyl)(1-phenylimidazo[1,5-a]pyridin-3-yl)methanone (**3q**)*

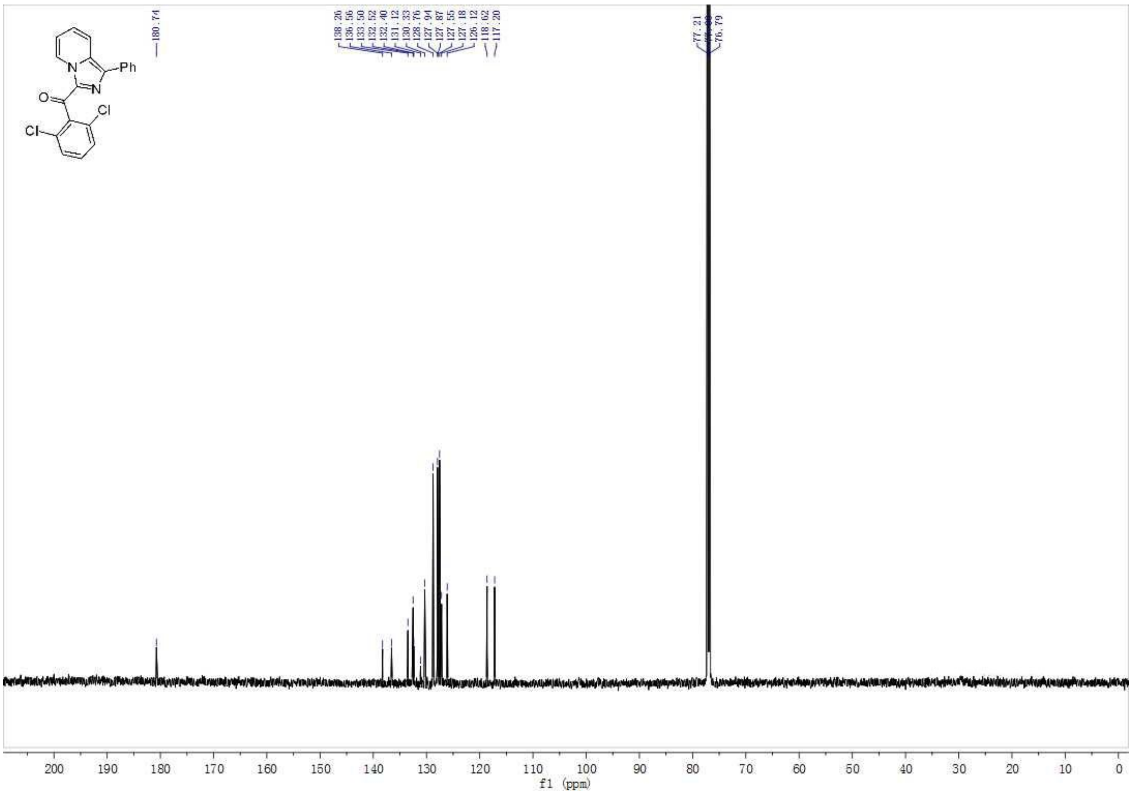

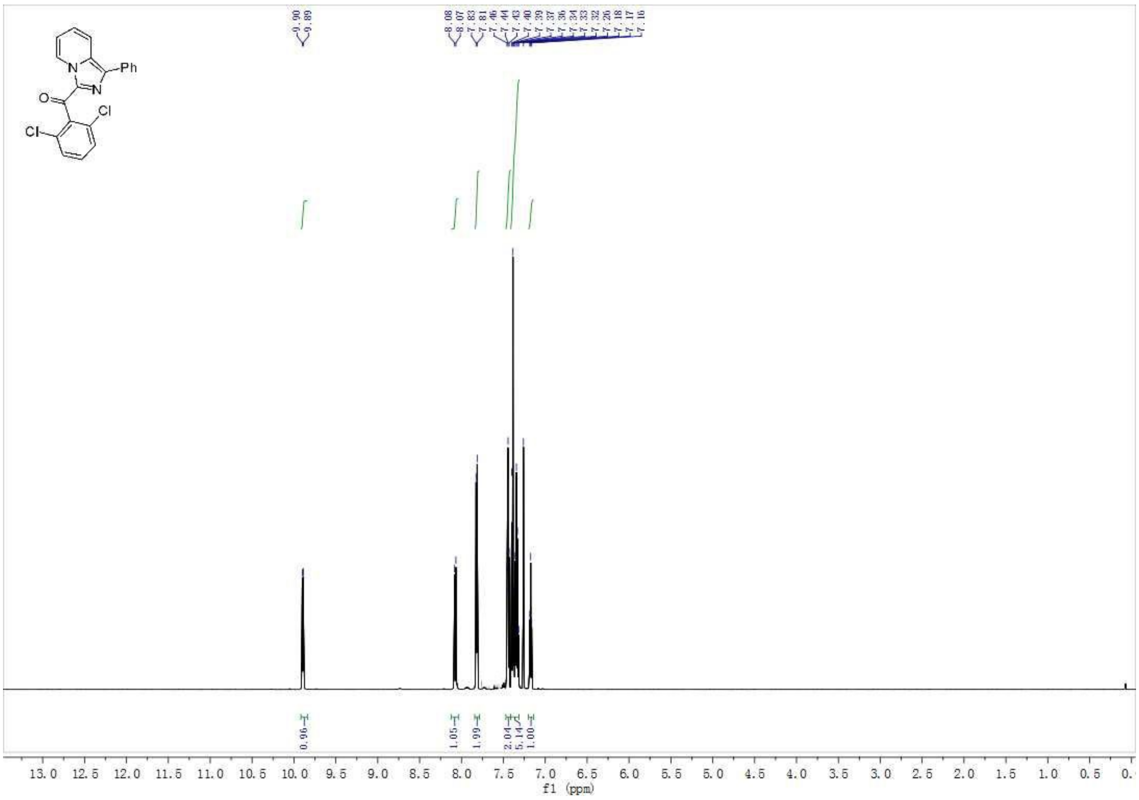

*(2,4-difluorophenyl)(1-phenylimidazo[1,5-a]pyridin-3-yl)methanone (**3r**)*

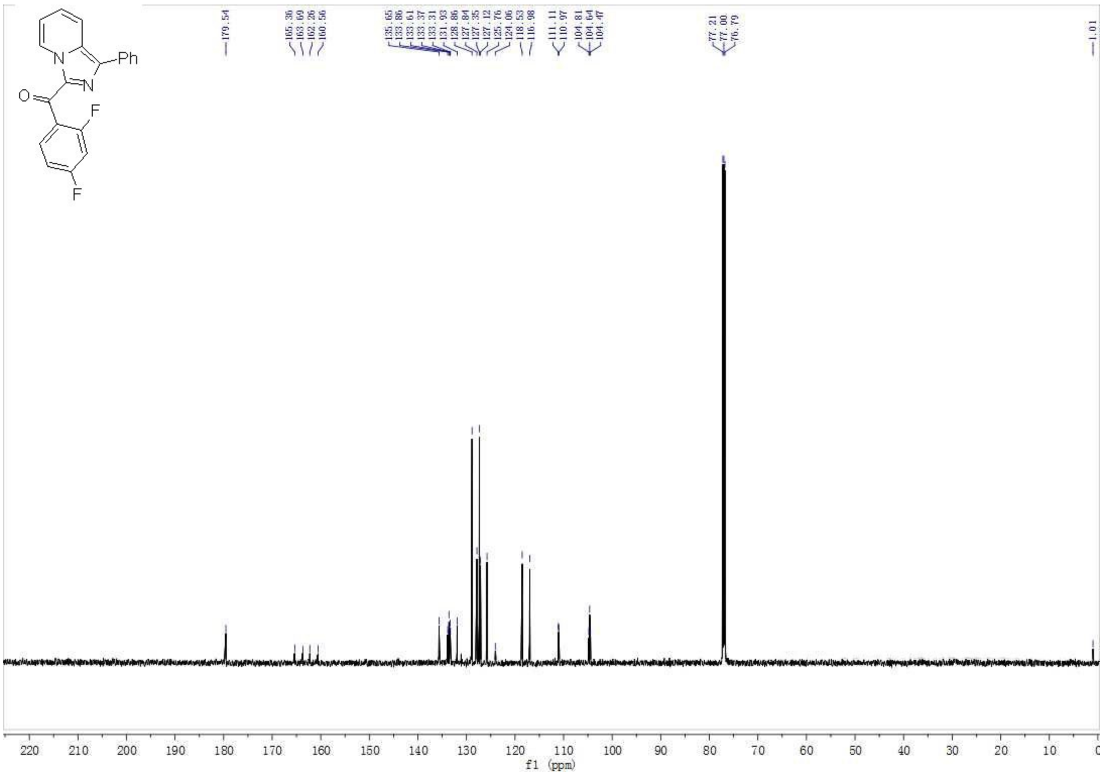

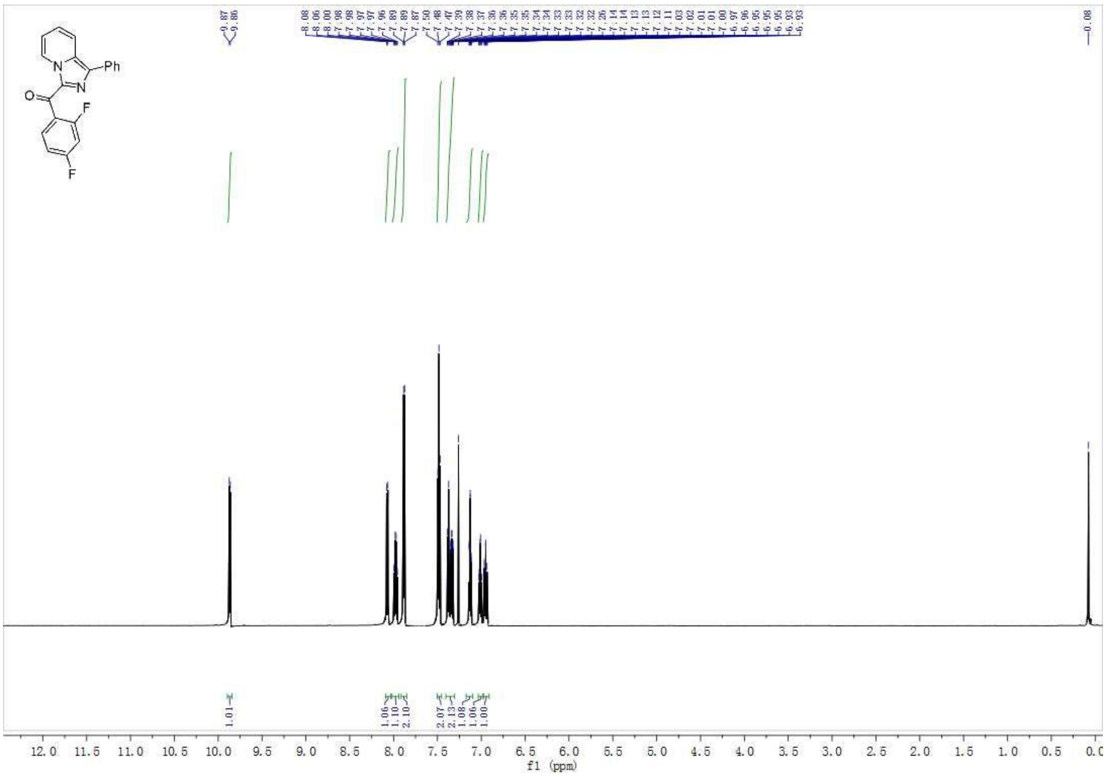

*(1-phenylimidazo[1,5-a]pyridin-3-yl)(2,4,5-trifluorophenyl)methanone (**3s**)*

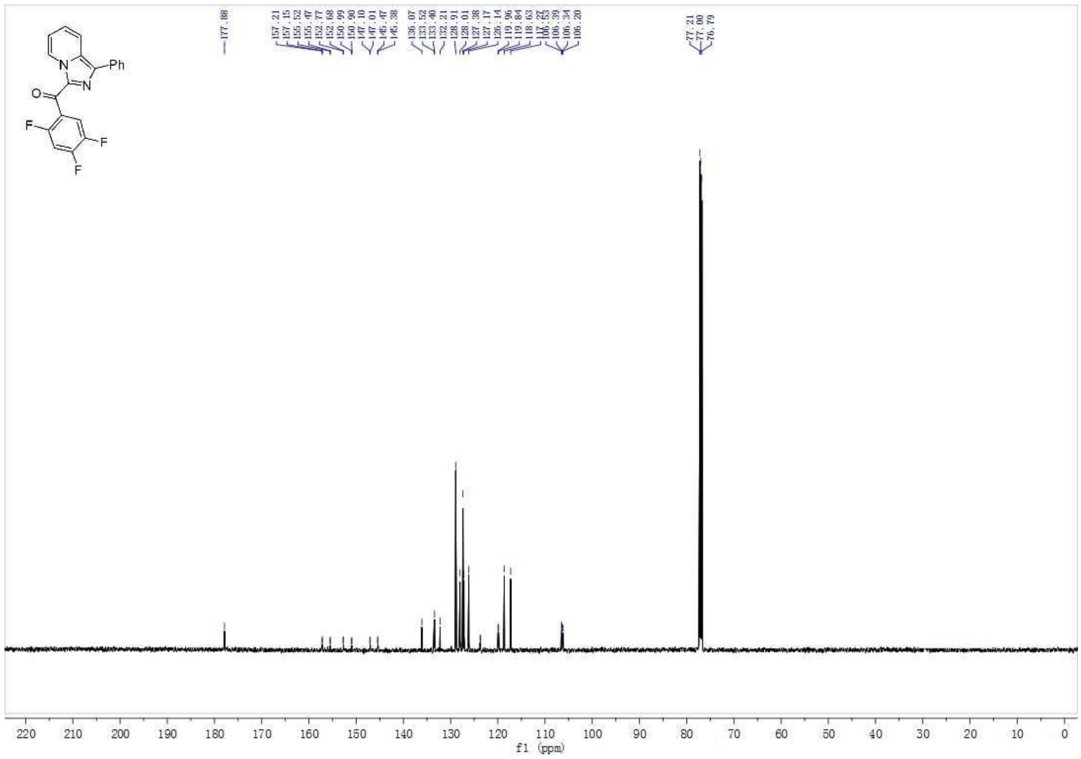

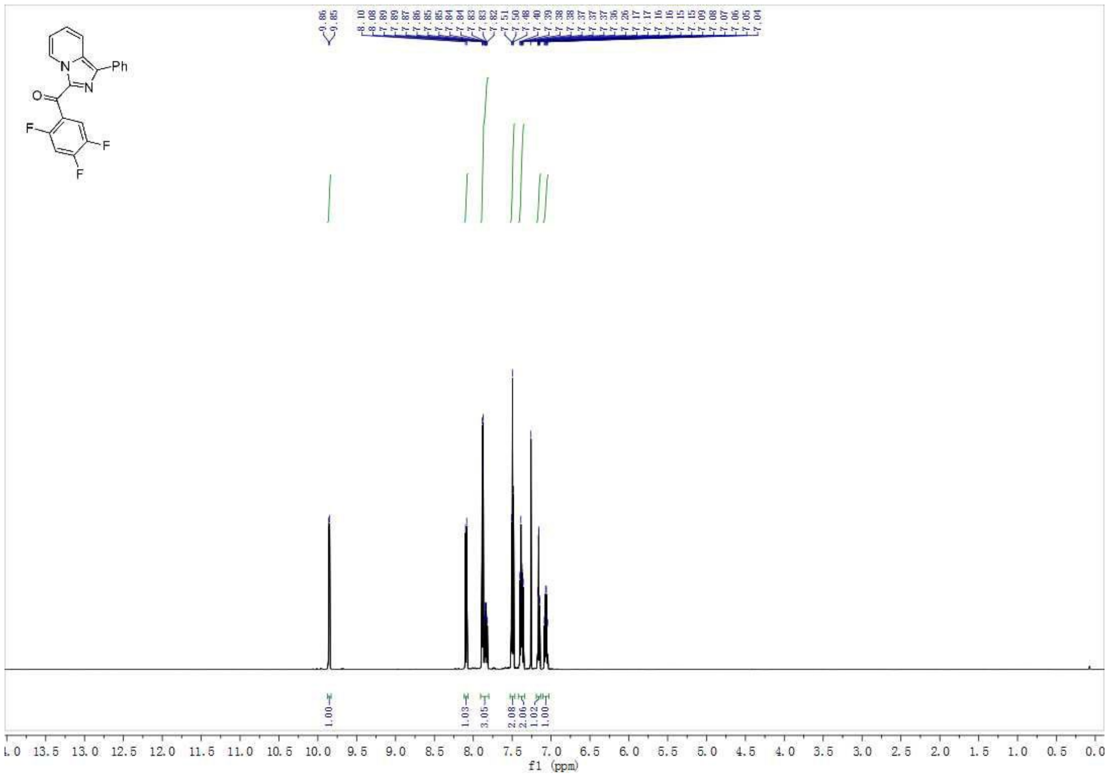

*(1-phenylimidazo[1,5-a]pyridin-3-yl)(pyridin-3-yl)methanone (**3t**)*

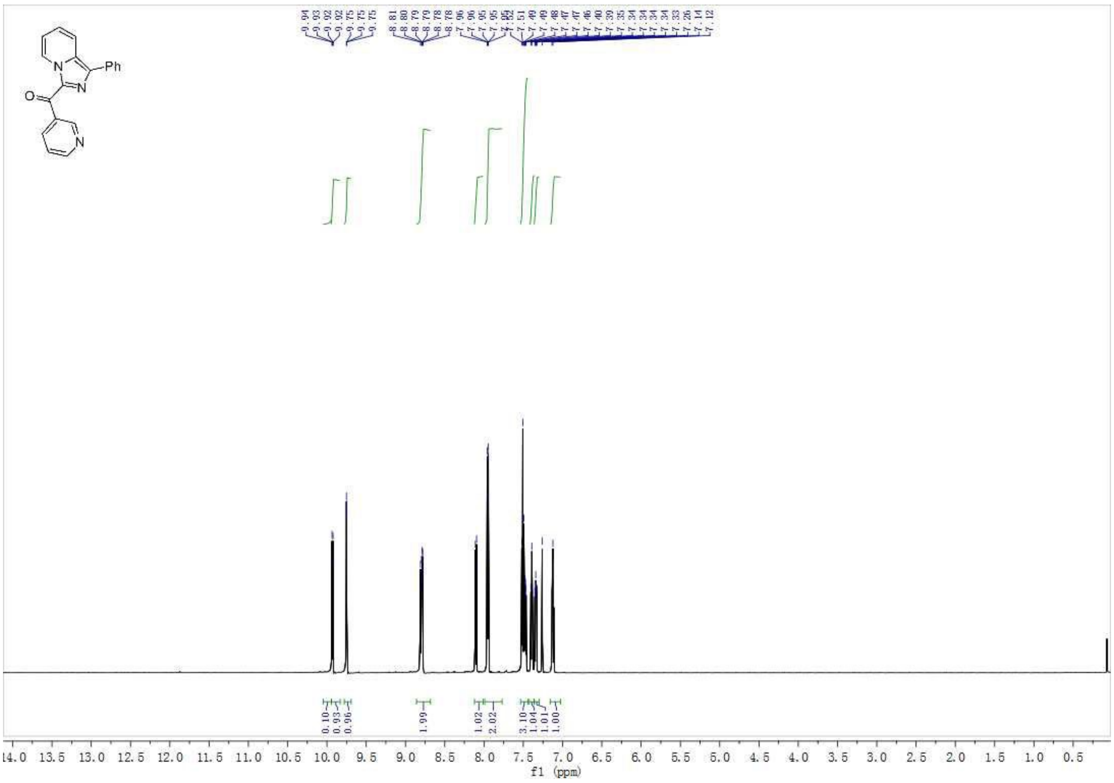

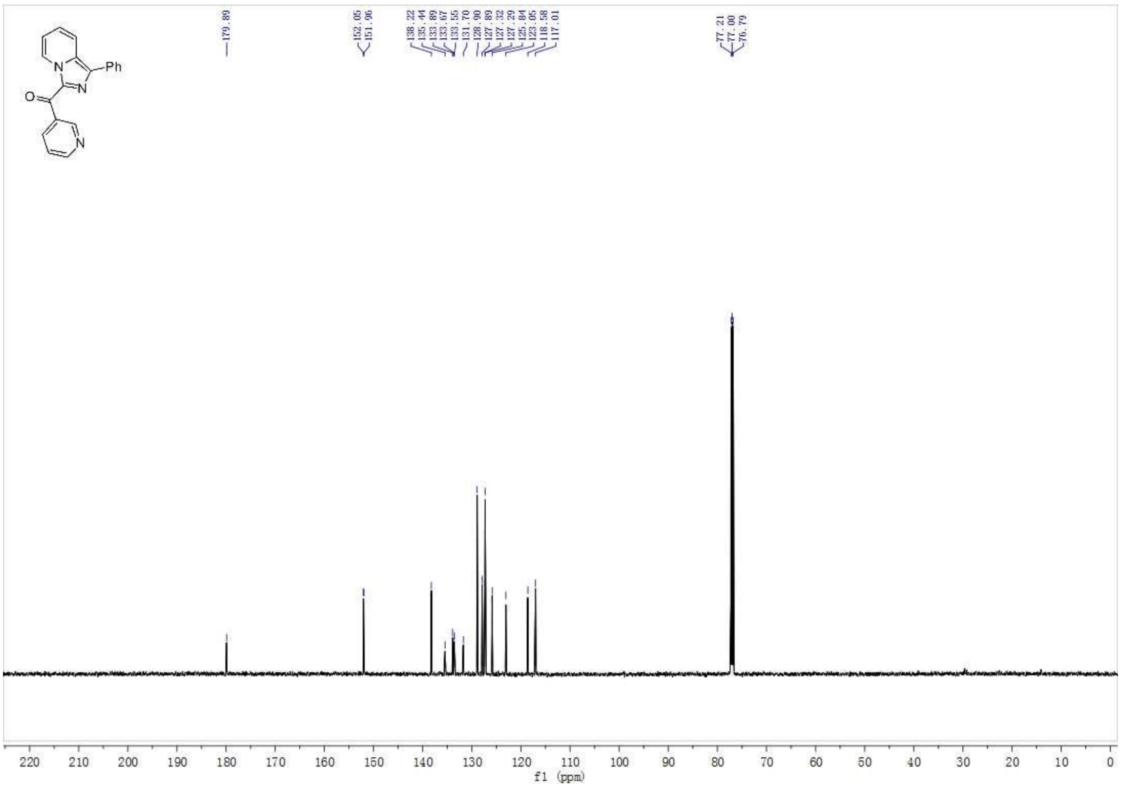

2. Procedure of detection of crucial intermediates by LC-MS

Sample preparation

To a solution of ethyl 3-oxo-3-phenylpropanoate (0.2 mmol, 1 equiv.), phenyl(pyridin-2-yl)methanamine (0.25 mmol, 1.25 equiv.) in 2mL of DMSO in a tube was added I_2 (0.6 mmol, 3equiv.) and H_2O (0.6 mmol), and the tube was heated at 75℃ and for 2 hours with oil bath. The system was cooled to room temperature, water (10 mL) was added and the aqueous solution was extracted with ethyl acetate (3 x 30 mL), the combined organic phase was washed with saturated brine and concentrated under reduced pressure, the residue was dissolved by MeOH and tested by LC-MS (Instrument: Waters HClass).

LC-MS detecting condition: Mobile phase A: 0.1% formic acid in H_2O; B: 0.1% formate in acetonitrile. Chromatographic column: waters BEH C18 1.7um 2.1*50mm. Column temperature: 40℃.

Time (min)	Flow rate（mL/min）	A(%)	B(%)
0	0.4	95	5
1	0.4	95	5
7	0.4	2	98
10	0.4	2	98

Results and explanations

II and **III** are isomeride, target molecular ion peak appeared twice at two different retention time would be reasonable. **II** and **III** were indeed detected at retention time of 2.47 and 4.38. m/z [M+H$^+$] calcd for $C_{23}H_{20}N_2O_3$ (**II** and **III**), 373.1552, found 373.1546 and 373.1548. This evidence can only to some extent explain the rationality of mechanism, for the isomerides of **II** and **III** are uncertain.

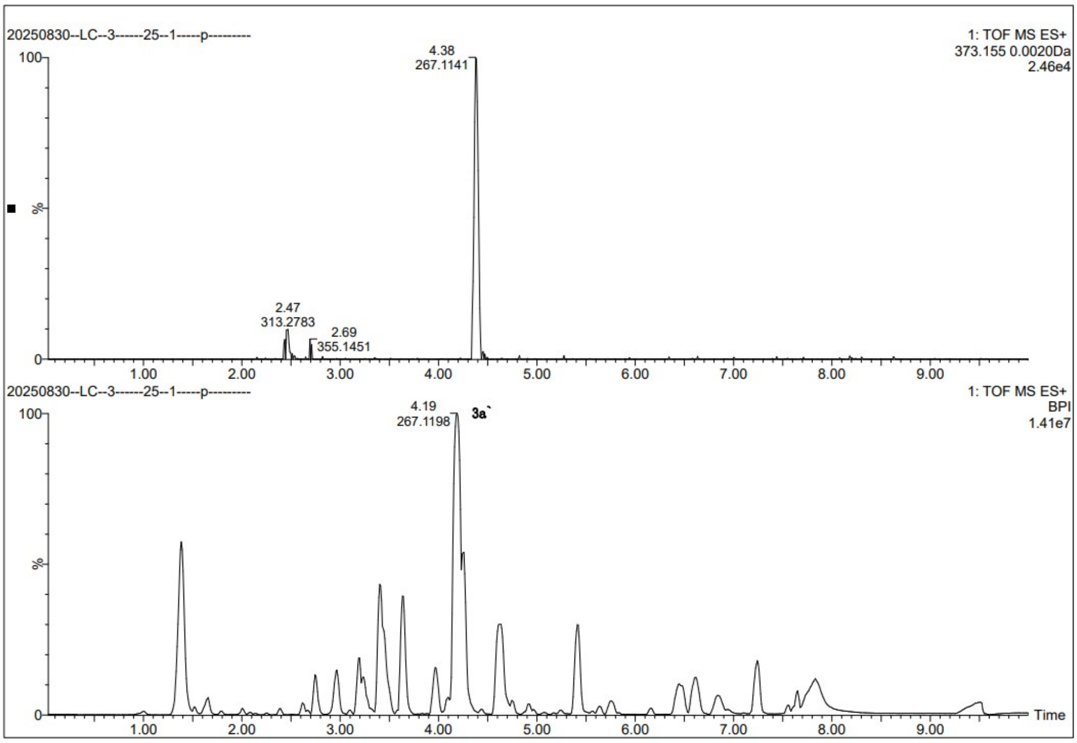

(1) Retention time of 2.47, m/z found 373.1546

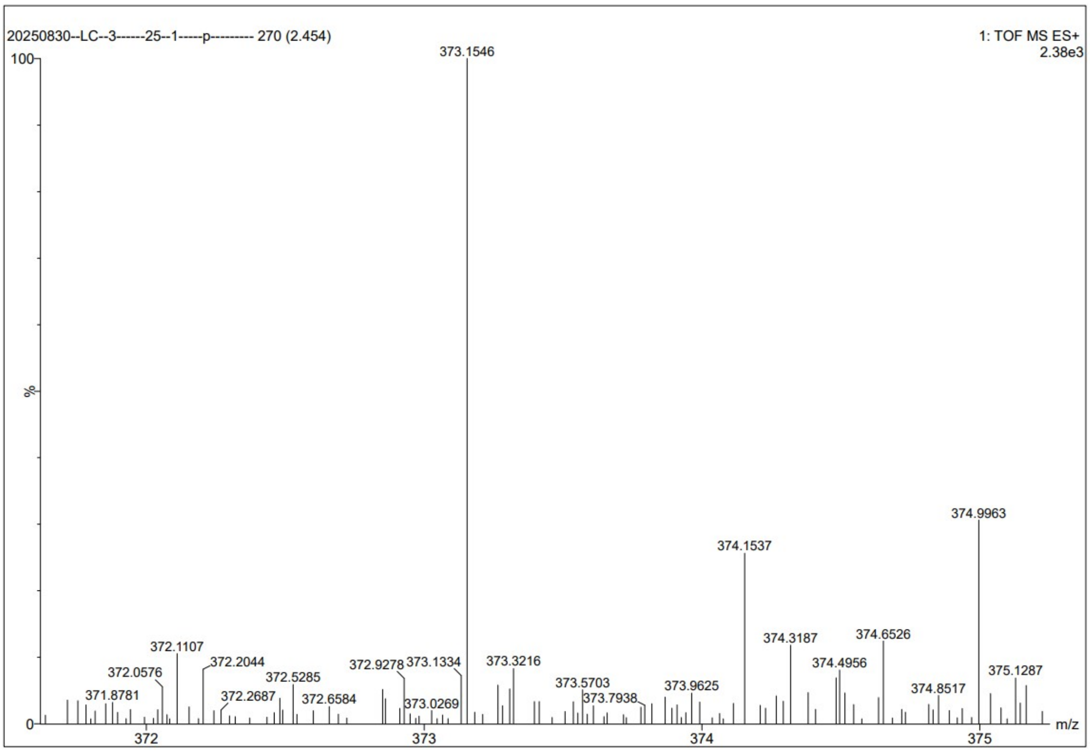

(2) Retention time of 4.38, m/z found 373.1548

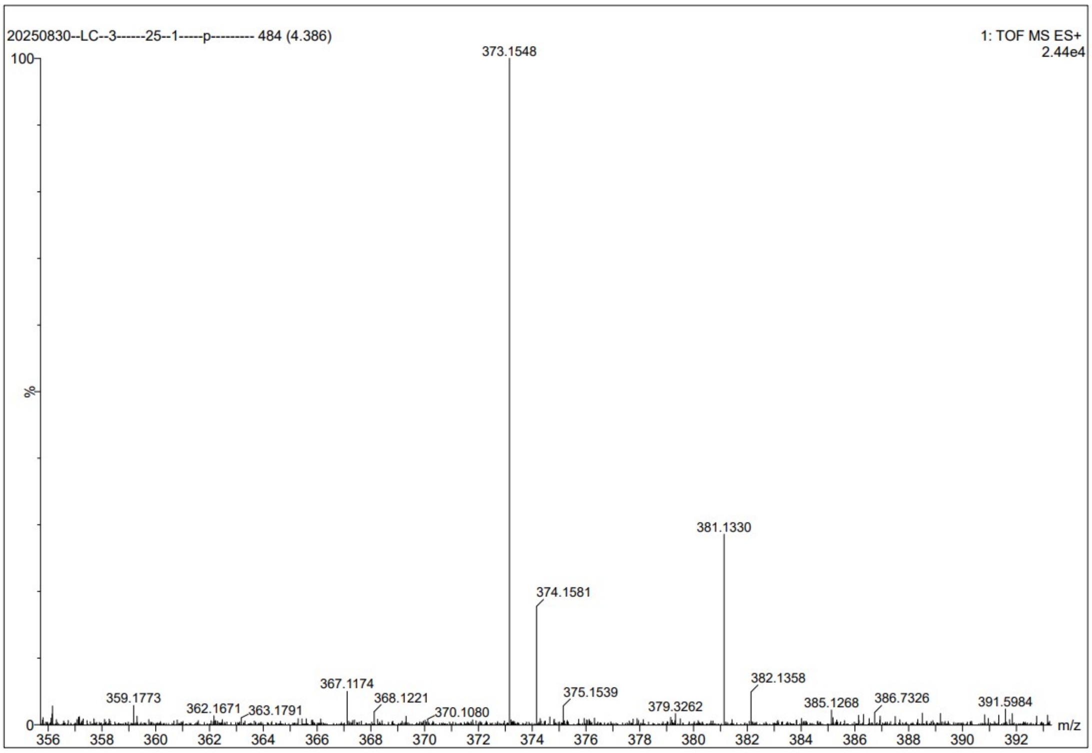

Supplement: RA-015-D5RA07797D-s001 [file RA-015-D5RA07797D-s001.pdf]
